# *Fragaria vesca* CONSTANS controls photoperiodic flowering and vegetative development

**DOI:** 10.1093/jxb/erx301

**Published:** 2017-09-25

**Authors:** Takeshi Kurokura, Samia Samad, Elli Koskela, Katriina Mouhu, Timo Hytönen

**Affiliations:** 1 School of Biological Sciences, University of Reading, Reading, Berkshire RG6 6AS, UK; 2 Department of Agricultural Sciences, Viikki Plant Science Centre, University of Helsinki, PO Box 27, FIN-00014 Helsinki, Finland; 3 Faculty of Agriculture, Utsunomiya University, Tochigi, 321-8505, Japan; 4 Department of Biosciences, Viikki Plant Science Centre, University of Helsinki, PO Box 56, FIN-00014 Helsinki, Finland

**Keywords:** CONSTANS, FLOWERING LOCUS T, *Fragaria*, photoperiod, reproduction, runner, strawberry

## Abstract

According to the external coincidence model, photoperiodic flowering occurs when *CONSTANS* (*CO*) mRNA expression coincides with light in the afternoon of long days (LDs), leading to the activation of *FLOWERING LOCUS T* (*FT*). CO has evolved in Brassicaceae from other Group Ia CO-like (COL) proteins which do not control photoperiodic flowering in Arabidopsis. COLs in other species have evolved different functions as floral activators or even as repressors. To understand photoperiodic development in the perennial rosaceous model species woodland strawberry, we functionally characterized FvCO, the only Group Ia COL in its genome. We demonstrate that FvCO has a major role in the photoperiodic control of flowering and vegetative reproduction through runners. FvCO is needed to generate a bimodal rhythm of *FvFT1* which encodes a floral activator in the LD accession Hawaii-4: a sharp *FvCO* expression peak at dawn is followed by the *FvFT1* morning peak in LDs indicating possible direct regulation, but additional factors that may include FvGI and FvFKF1 are probably needed to schedule the second *FvFT1* peak around dusk. These results demonstrate that although FvCO and FvFT1 play major roles in photoperiodic development, the *CO*-based external coincidence around dusk is not fully applicable to the woodland strawberry.

## Teaser

Woodland strawberry (*Fragaria vesca*) contains only one Group Ia CONSTANS-LIKE protein. It controls vegetative and reproductive development and activates *FT*, but additional factors are needed to generate rhythmic *FT* expression.

## Introduction

Plants use various environmental cues, such as light and temperature, to synchronize their life cycles according to local climate (Yanovsky and Kay, 2003). The external coincidence model indicates how environment is linked to flowering in Arabidopsis (Salomé and McClung, 2004; Nozue *et al.*, 2007). According to this model, flower induction takes place when external stimuli such as photoperiod meet with the active phase of an internal oscillator (Sawa *et al.*, 2008). A small transcription factor CONSTANS (CO) is at the heart of the external coincidence model (Suárez-López *et al.*, 2001; Valverde *et al.*, 2004): photoperiodic flowering occurs when the *CO* mRNA expression peaks at the end of the light period in long days (LDs) and CO activates the expression of *FLOWERING LOCUS T* (*FT*) that encodes a mobile flowering-inducing signal (Corbesier *et al.*, 2007; Tamaki *et al.*, 2007).

The circadian clock indirectly generates the rhythmic expression of *CO*. The basic mechanism of this clock involves feedback loops of genes that are expressed in different phases of the daily cycle (McClung, 2009). The core feedback loop, formed by *TIMING OF CAB EXPRESSION* (*TOC1*) and *LATE ELONGATED HYPOCOTYL/CIRCADIAN CLOCK ASSOCIATED1* (*LHY/CCA1*) (Yanovsky and Kay, 2003; Más, 2008; McClung, 2008), generates the rhythmic expression of several flowering time regulators including FLAVIN-BINDING, KELCH REPEAT, AND F-BOX 1 (FKF1), GIGANTEA (GI), and CYCLING DOF FACTOR1 (CDF1) (Huq *et al.*, 2000; Mizoguchi *et al.*, 2005; Fornara *et al.*, 2009). In the morning, CDF1 directly binds to the promoter of *CO* to suppress the transcription of the gene (Imaizumi *et al.*, 2005). CDF is degraded by the GI–FKF1 protein complex during the day, leading to a peak in *CO* expression in the afternoon (Sawa *et al.*, 2007).

Along with transcriptional regulation, CO protein concentration is also strictly regulated by light. CO is unstable in darkness and in the morning, when E3 ubiquitin ligase HIGH EXPRESSION OF OSMOTICALLY RESPONSIVE GENES1 (HOS1) and phytochrome B (PHYB) activated by red light together destabilize CO (Valverde *et al.*, 2004; Lazaro *et al.*, 2015). In the afternoon, however, phytochrome A and cryptochrome 2 (PHYA and CRY2), which respond to far-red and blue light, respectively, stabilize CO. Therefore, CO protein only accumulates under LD conditions when *CO* mRNA expression peaks during the light period. This results in the activation of *FT* and thus flowering. On the other hand, under short-day (SD) conditions, *CO* mRNA expression peaks in the middle of the night, when CO protein cannot accumulate sufficiently to activate *FT* (Blázquez and Weigel, 1999; Suárez-López *et al.*, 2001; Izawa *et al.*, 2002; Valverde *et al.*, 2004; Endo *et al.*, 2005).

CO has two B-box type zinc finger domains which have been proposed to function in protein–protein interaction (Putterill *et al.*, 1995; Robson *et al.*, 2001). It also has one CCT (CO, CO-like, TOC1) domain on its C-terminus which mediates protein–protein interaction and nuclear localization (Robson *et al.*, 2001; Ben-Naim, 2006; Wenkel *et al.*, 2006). A total of 16 *CO* homologous genes, all of them with at least one B-box domain and one CCT domain, have been isolated from Arabidopsis and designated as *CO*-like (*COL*) *1–16* (Robson *et al.*, 2001; Griffiths *et al.*, 2003). These genes were allocated to Groups I–III, according to the degree of conservation and number of B-box domains (Robson *et al.*, 2001). Group I, which includes the *CO* gene, was subdivided into Ia–Id according to the extent of conservation of four highly conserved regions in the middle (Griffiths *et al.*, 2003). A recent study has provided evidence that *COL1* and *COL2*, that do not encode floral promoters, are ancestral Group Ia *COL* genes; the floral promoter CO evolved within the Brassicaceae after the family split from the Cleomaceae (Simon *et al.*, 2015). In addition to distinct functions, these *COL* genes show the highest expression at dawn, in contrast to *CO* which peaks in the afternoon (Ledger *et al.*, 2001; Simon *et al.*, 2015).

*CO* homologues have been isolated from other plants including woody plants, monocotyledons, and even single-celled *Chlamydomonas* (Song *et al.*, 1998; Lagercrantz and Axelsson, 2000; Yuceer *et al.*, 2002; Griffiths *et al.*, 2003; Nemoto *et al.*, 2003; Chia *et al.*, 2008; Holefors *et al.*, 2009; Serrano *et al.*, 2009). In the SD plant rice (*Oryza sativa*), the *CO* homologue *Heading date1* (*Hd1*) promotes expression of the *FT* homologue *Hd3a* under inductive SDs (Izawa *et al.*, 2002; Ishikawa *et al.*, 2005). Other *CO* homologues, *OsCO3* (*OsB*) and *OsCOL10*, have a negative effect on the expression of *Hd3a* under these conditions (Kim *et al.*, 2008; Tan *et al.*, 2016). Several *CO*-like genes have also been identified in *Chrysanthemum* spp., and one of these was found to promote *FT* expression and flowering (Fu *et al.*, 2015). However, studies in *Pharbitis nil* and *Medicago truncatula* indicated that their *COL* genes are not involved in the control of *FT* expression and flowering (Hayama *et al.*, 2007; Wong *et al.*, 2014).

FT has been shown to function as a floral activator in SD, LD, and day-neutral plants, while another member of the same gene family, TERMINAL FLOWER1 (TFL1), is a floral repressor (Wickland and Hanzawa, 2015). However, there are several independent examples about the evolution of FT homologues into floral repressors including BvFT1 in sugar beet, three FT homologues in tobacco, and a specific splicing variant of *Brachypodium* FT (Pin *et al.*, 2010; Harig *et al.*, 2012; Qin *et al.*, 2017). In seasonal flowering commercial strawberry (*Fragaria*×*ananassa* Duch.) and the diploid model woodland strawberry (*Fragaria vesca* L.), which are both SD plants (Ito and Saito, 1962; Battey *et al.*, 1998; Battey, 2000), TFL1 homologues are strong floral repressors. *FvTFL1* and *FaTFL1* are highly expressed under LDs, and their repression under SDs and low temperature conditions enables flower induction to take place (Koskela *et al.*, 2012; Nakano *et al.*, 2015; Rantanen *et al.*, 2015; Koskela *et al.*, 2016). Interestingly, *FT* homologues, *FvFT1* and *FaFT1*, are expressed specifically under LDs and correlate negatively with flower induction, indicating that they may also repress flowering in SD strawberries (Koskela *et al.*, 2012; Nakano *et al.*, 2015). A natural mutant of woodland strawberry (*F. vesca semperflorens*) lacks functional FvTFL1 and is an LD plant which flowers perpetually after flower induction (Koskela *et al.*, 2012). In this mutant, *FvFT1* is also expressed under LDs and functions as a promoter of flowering (Koskela *et al.*, 2012; Rantanen *et al.*, 2014). *FvFT1* is normally expressed diurnally with peaks 4 h and 16 h after dawn; its expression is most effectively induced artificially by FR daylength extension in the mutant. (Koskela *et al.*, 2012; Rantanen *et al.*, 2014).

A close *CO* homologue (*FvCO*) has been previously identified in woodland strawberry (Shulaev *et al.*, 2011), but its function has not been tested. Here, using transgenic overexpression and RNAi lines of woodland strawberry, we demonstrate that *FvCO* has a major role in the photoperiodic development of this species. We show that, although the gene expression rhythms of *FvCO* and *FvFT1* do not coincide, *FvCO* is needed to activate *FvFT1* that controls reproductive and vegetative development in response to photoperiodic signals.

## Materials and methods

### Plant material

Experiments were mostly performed with the LD-flowering accession ‘Hawaii-4’ (H4; National Clonal Germplasm Repository accession number PI551572). Gene expression analyses were also carried out in a Finnish SD accession FIN56 (PI551792). Seedlings or plants clonally propagated from runner cuttings were used for the experiments as indicated in the text and figure legends.

### Growth conditions and phenotypic observations

Plants were raised in a growth chamber or a greenhouse under a non-flower-inductive photoperiod at 20–22 °C, under SDs (12/12 h light/dark) for H4 and LDs (16/8 h light/dark) for FIN56. Fluorescent tubes (Warm white 30W/32-930, Osram, Germany) or light-emitting diodes (LEDs; AP67, Valoya, Finland) were used as the white light source at a photosynthetic photon flux density (PPFD) of 200 µmol m^–2^ s^–1^ in growth chambers. High pressure sodium (HPS) lamps (Airam 400W, Kerava, Finland) at a PPFD of 120 µmol m^–2^ s^–1^ were used to supplement the natural light in the greenhouse. Seedlings were transplanted to 8 × 8 cm pots at the cotyledon stage, while runner cuttings were directly rooted in these pots. Fertilized peat (Kekkilä, Finland) supplemented with 25% (v/v) vermiculite (Ø2 mm) was used as a growing medium. Plants were fertilized with liquid fertilizer (Kekkilä; N-P-K: 17-4-25) biweekly.

Both flowering time and vegetative development were studied in the experiments. To observe flowering time differences between H4 and transgenic lines, either the number of leaves in the primary leaf rosette before the terminal inflorescence or the number of days before the first open flower was recorded. In addition, the differentiation of axillary buds into either axillary leaf rosettes called branch crowns or runners (stolons) was observed.

### Gene expression analysis

Leaf and shoot apex samples were frozen in liquid nitrogen and stored at –80 °C before total RNA was extracted using a modified cetyltrimethylammonium bromide (CTAB) method as described in Koskela *et al.* (2012). cDNAs were synthesized from 1 μg of total RNA using Superscript III reverse transcriptase (Invitrogen). SYBR Green I master mix (Roche) was used for real-time PCRs which were performed in the Light Cycler 480 instrument (Roche) as described previously (Mouhu *et al.*, 2009). Real-time PCR reactions were performed with three technical replicates and two or three biological replicates as mentioned in the figure legends. Relative expression levels were calculated by the ∆∆Ct (cycle threshold) method, with *FvMSI1* as the normalization gene as described previously (Mouhu *et al.*, 2013). Primers used in the real-time PCR are listed in Supplementary Table S1 at *JXB* online. Primer efficiencies were almost equal for all primer pairs (Rantanen *et al.*, 2014).

### Plasmid constructs

Plasmid constructs for overexpression and RNAi silencing lines were created according to Gateway technology with Clonase II (Invitrogen). For *FvCO* overexpression and RNAi constructs, cDNA from *F. vesca* H4 was amplified with primer pairs 5Y76J-(attB1)-TGAGAGTGAGGAGGAAACAACA-3' and 5'-(attB2)-TTGCTGCAAAAGGTTGAACT-3', and 5'-(attB1)-ACAATCCGGTATGCCTCAAG-3' and 5-(attB2)-AGGAACAATGCCGTATCCAG-3', respectively. The destination vectors were pK7WG2D.1 for overexpression and pK7GWIWG2D(II) for RNAi silencing (Karimi *et al.*, 2002). Both vectors contain green fluorescent protein as a positive selection marker.

### Transformation

Vectors carrying overexpression and RNAi constructs were electroporated into *Agrobacterium tumefaciens* strain GV3101 and transformed into H4 as described previously (Oosumi *et al.*, 2006). Several transgenic lines were generated for both constructs. Transgenic lines were selected for the experiments based on their phenotypes and *FvCO* expression levels.

### Sequence alignment and phylogenetic analysis

Amino acid sequence alignment was conducted using the ClustalW program with the BLOSUM62 matrix. MrBayes 3.2.2 was used to construct a Bayesian estimation of a phylogeny of CO-like proteins. Two independent runs were performed, the averaged. WAG (Whelan and Goldman) matrix was used as a substitution model, and gamma distribution was set for among-site rate variation with the rate category of 4. The Markov chain Monte Carlo algorithm was run with chain length of 1 000 000 with four heated chains (heated chain temperature=0.2). Subsampling was performed every 200 generations and burn-in length was set to 10%. CrCO from *Chlamydomonas reinhardtii* was used as the outgroup.

### Statistical analyses

ANOVA was conducted on the averages using the general linear model, and differences between means were analysed by Tukey–Kramer test. All statistical analyses were conducted using the R package (ver. 3.3.2).

### Accession numbers

Sequence data from this article can be found in the GenBank/National Center for Biotechnology Information data library under the following accession numbers: *FvSOC1* (FJ531999) and *FvFT1* (JN172098). Predicted gene models (Hybrid V2) can be found in the Genome Database for Rosaceae (http://www.rosaceae.org): *FvCO* (gene04172), *FvMSI1* (gene03001), gene02008, gene03742, gene14015, gene14981, gene15552, gene24941, gene25039, gene25171, and gene27383. Accession numbers of the protein sequences used in the phylogenetic analysis are listed in Supplementary Table S2.

## Results

### Isolation, structure, and phylogenetic analysis of *Fragaria CO*

One woodland strawberry homologue of *CO*, *FvCO* (gene04172), was previously annotated in the *F. vesca* whole-genome v1.1 assembly (Shulaev *et al.*, 2011). To explore the strawberry *CO*-like gene family, a BLASTx database search was performed using the full-length sequence of *FvCO* against the whole-genome assembly. In total, nine additional putative CO-like protein sequences longer than 200 amino acids were identified. These protein sequences were subjected to a phylogenetic analysis to identify putative regulators of flowering time.

**Fig. 1.**
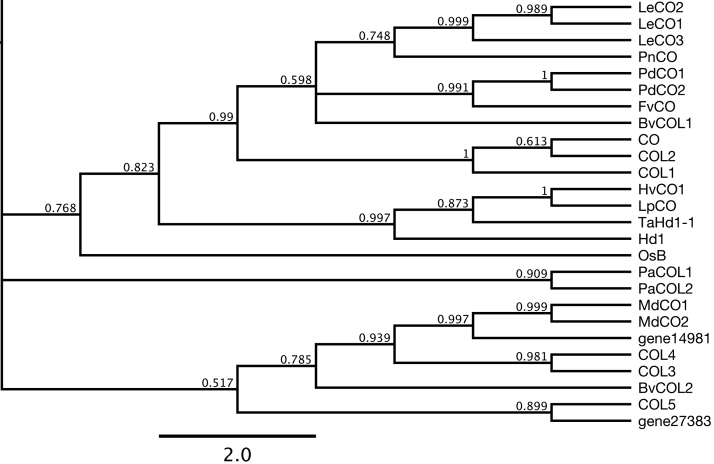
A phylogenetic tree of COL proteins from woodland strawberry and other species. A part of the phylogenetic tree containing Group I genes is shown. The full tree structure is available as Supplementary Fig. S1. The list of species and protein accessions is available in Supplementary Table S2. Numbers on each node indicate posterior probabilities.

A phylogenetic tree of COL proteins was constructed using CrCO from *C. reinhardtii* as the outgroup. FvCO was placed in the same clade with CO homologues of eastern cottonwood, morning glory, and tomato, and with Arabidopsis Group Ia proteins CO, COL1, and COL2 (Fig. 1; Supplementary Fig. S1). The predicted protein for gene14981 was placed in the clade comprised of *Malus domestica* CO-like proteins, BvCOL2 of sugar beet, and Arabidopsis COL3 and COL4, which are categorized as CO Group Ib proteins (Griffiths *et al.*, 2003; Chia *et al.*, 2008); the predicted protein for gene27383 was close to COL5 (Fig. 1; Supplementary Fig. S1). Other predicted proteins clustered in Group II (gene03742 and gene25171) or Group III (gene14015, gene15552, and gene24941); gene02008 and gene25039 made up an isolated clade of their own (see Supplementary Fig. S1).

As the phylogenetic tree indicated that *FvCO*, gene14981, and gene27383 belong to Group I, conserved domains of the corresponding protein sequences were subjected to further analysis (Supplementary Fig. S2a). Predicted protein sequences of these genes were aligned with other CO-like proteins by ClustalW. The alignment showed that two B-box domains (Griffiths *et al.*, 2003) and the CCT domain (Wenkel *et al.*, 2006) were highly conserved in these three *Fragaria CO*-like sequences (Supplementary Fig. S2b, c). FvCO showed the highest level of conservation in the M1–M4 conserved regions. Gene27383 had glutamate to aspartate, tryptophan to leucine, and leucine to isoleucine substitutions in the M1 region found in Group Ia–Ic (Glu-X_2_-Ser-Trp-Leu-Leu), while the other two have a conserved sequence (Supplementary Fig. S2d). The M2 region (Leu-Val-Asp/Gly-Tyr) of FvCO had an aspartate/glycine to glutamate substitution similarly to PnCO (Group Ia), and the other two were lacking valine similarly to COL3 and COL4 (Group Ic) (Supplementary Fig. S2e). The M3 region (Gly-X-Asp/Glu-X-Ile/Val-Val-Pro) of gene14981 had a substitution of the first glycine residue to alanine (Supplementary Fig. S2f), and the M4 region (Ser-X-Glu/Asp-X_3_-Val-Pro) of gene14981 had a substitution of the first serine to proline (Supplementary Fig. S2g). As the phylogenetic tree and further analyses on conserved domains indicated that FvCO was the only Group Ia COL protein encoded by the accessible woodland strawberry genome, functional analysis was mainly focused on FvCO.

### *FvCO* expression peaks at dawn

**Fig. 2.**
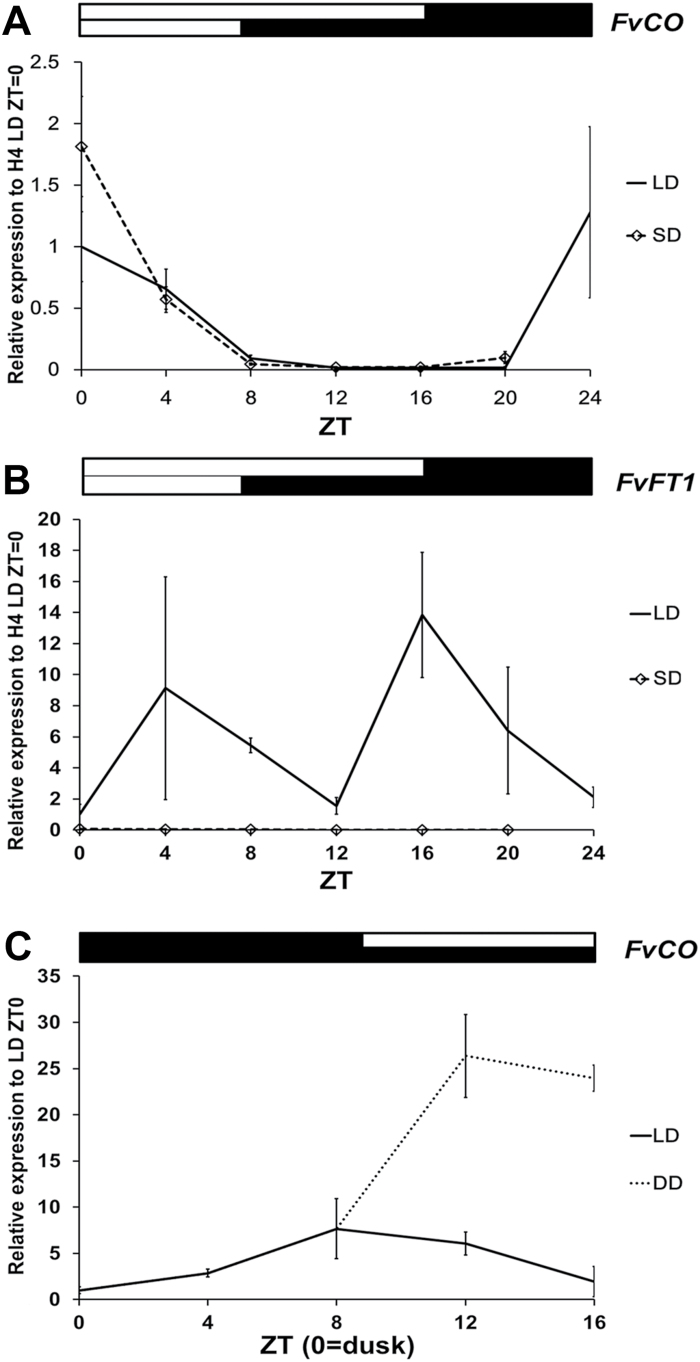
Expression patterns of *FvCO* and *FvFT1*. mRNA expression patterns of *FvCO* (A) and *FvFT1* (B) were analysed in the leaf samples of short day- (SD) and long day- (LD) grown H4 plants. FvCO mRNA expression was also analysed in plants moved from LDs to darkness (DD) (C). White and black bars above the panels indicate light and dark periods, respectively. The average expression level of three biological replicates is shown for each time point, all normalized to the expression level of *FvMSI1*. Error bars indicate the SD.

The diurnal expression patterns of *FvCO* and *FvFT1* were investigated in woodland strawberry accessions with contrasting photoperiodic responses. In the perpetual flowering LD accession H4and the seasonal flowering SD accession FIN56, *FvCO* exhibited a single mRNA expression peak at dawn under both LD and SD conditions, and its expression stayed low during the day regardless of the accessions examined (Fig. 2A; Supplementary Fig. S3). In H4 under LDs, the expression of *FvFT1* peaked 4–8 h after dawn and again in the evening (ZT16–ZT20), the second peak being slightly higher than the first (Fig. 2B). A similar diurnal rhythm of *FvFT1* mRNA expression was observed in FIN56 under LDs, but the morning peak was higher than the evening peak (Supplementary Fig. S3). The morning peak of *FvFT1* followed that of *FvCO*, but the evening peak of *FvFT1* did not coincide with high *FvCO* mRNA levels. In H4 under SDs, the *FvFT1* mRNA level was very low or undetectable throughout the 24 h cycle (Fig. 2B). In addition to *FvCO*, we explored the rhythmic expression of two *COL* genes showing the highest homology with *FvCO*. The gene27383 exhibited rhythmic expression, peaking at the same time as *FvCO*, whereas the expression of gene14981 did not show a clear rhythm (Supplementary Fig. S4).

Our data indicated that *FvCO* expression peaked at dawn under different photoperiods, so we tested whether the dawn signal was critical for the timing of its expression. LD-grown plants were transferred to darkness (DD) and *FvCO* mRNA levels measured. Under DD conditions, in contrast to the LD control, *FvCO* expression continued to rise after the subjective dawn (the beginning of the light period in the LD control) and stayed high during the next 8 h (Fig. 2C). These results suggest that the up-regulation of *FvCO* takes place in darkness and the dawn signal is needed for its down-regulation.

### FvCO controls vegetative and generative development

**Fig. 3.**
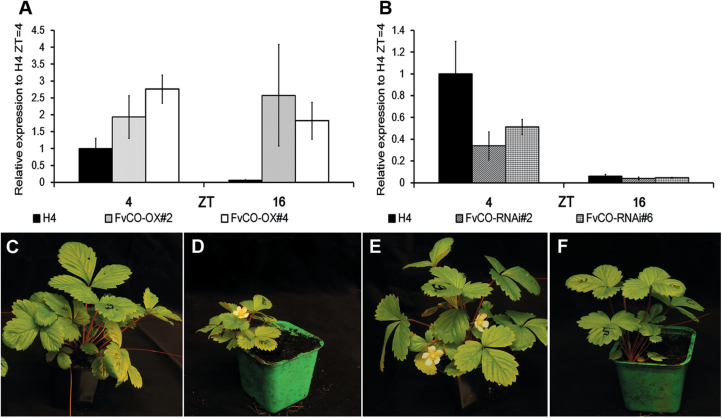
Flowering phenotypes of *FvCO* transgenic plants. mRNA expression levels of *FvCO* were analysed in the leaf samples of overexpression (A) and RNAi lines (B) after plants were transferred to LD conditions for 2 weeks. Samples were taken at ZT4 and ZT16. The average expression level of three biological replicates is shown for each time point, all normalized to the expression level of *FvMSI1*. Error bars indicate the SD. (C–F) Flowering phenotypes of wild-type H4 (C, E), the overexpression line #2 (D), and the RNAi line #2 (F) after plants were placed under SD (C, D) or LD (E, F) conditions for 6 weeks.

To test the role of *FvCO* in the photoperiodic control of vegetative and reproductive development, we generated transgenic plants of the H4 accession with *FvCO* overexpressed [driven by the *Cauliflower mosaic virus* (CaMV) *35S* promoter] or RNAi silenced. The expression levels of *FvCO* mRNA were clearly altered in these transgenic lines (Fig. 3A, B). In the overexpression lines, strong up-regulation of *FvCO* was observed especially in the evening (ZT16) when its expression level in the wild-type H4 is low. In RNAi lines, in contrast, clear down-regulation of *FvCO* was observed, but no silencing of two other Group I *COL* genes was detected, confirming the specificity of our RNAi construct (Supplementary Fig. S4).

**Fig. 4.**
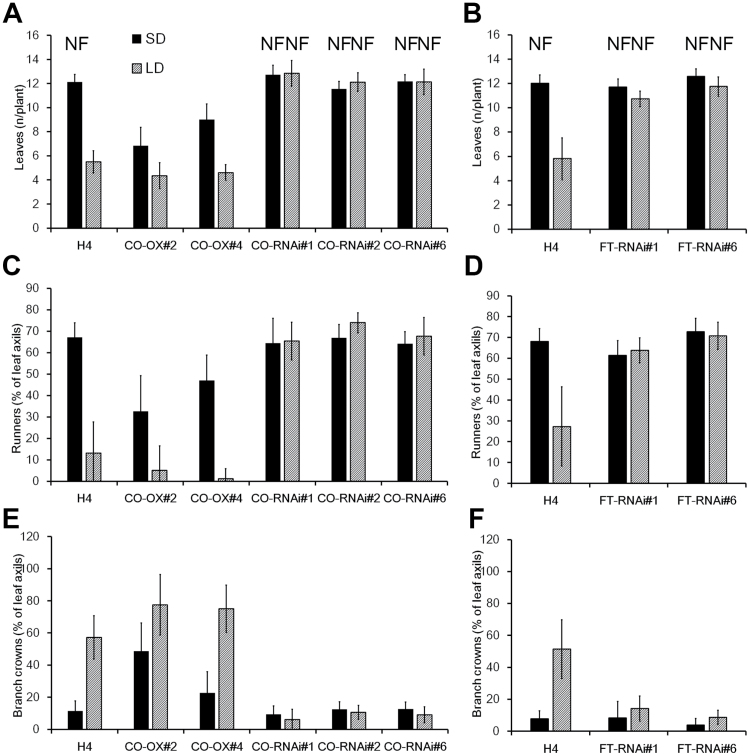
Vegetative and generative development in transgenic lines. Number of leaves emerged before the terminal inflorescence (A, B) and the percentage of axillary buds of the primary shoot differentiated to runners (C, D) or branch crowns (E, F) in *FvCO* transgenic lines (A, C, E) or in *FvFT* RNAi lines (B, D, F) Plants were grown under SD or LD conditions for up to 10 weeks (*n*=10). NF, no flowering. Axillary buds that did not differentiate to runners or branch crowns remained dormant.

We recorded the number of leaves in the primary leaf rosette before the terminal inflorescence in plants that had been subjected to LDs or SDs. Overexpression lines produced slightly fewer leaves before the terminal inflorescence compared with wild-type plants under LDs, whereas a strong promotion of flowering was observed in overexpression lines under SDs (Figs 3C, D, 4A; Supplementary Table S3). In *FvCO* RNAi lines, in contrast, flowering was significantly delayed compared with non-transgenic control plants under LDs, while under SDs, both H4 and *FvCO* RNAi lines remained vegetative or flowered very late, depending on the experiment (Figs 3E, F, 4A; Supplementary Table S3). An additional experiment revealed that *FvCO* overexpression plants flowered within 4 weeks and wild-type H4 after 5 weeks in LDs, whereas *FvCO* RNAi lines flowered ~1 month later (Supplementary Fig. S5). Comparison of *FvCO* RNAi lines with the previously published *FvFT1* RNAi lines (Koskela *et al.*, 2012) showed that both constructs had a similar effect on flowering time in H4 (Fig. 4B; Supplementary Table S3).

Flower-inducing conditions promote the differentiation of axillary buds to axillary leaf rosettes called branch crowns, while in non-inductive conditions vegetative reproduction through runners takes place. To gain insight into the effect of FvCO and FvFT1 on vegetative development, we studied the differentiation of axillary buds of the primary leaf rosette. In H4, most axillary buds differentiated to runners in SD conditions and only a few branch crowns were observed, whereas the effect of LDs was opposite (Fig. 4C–F). A clear photoperiodic response was also observed in *FvCO* overexpression lines, although they tended to produce fewer runners and more branch crowns than the wild type. In both *FvCO* and *FvFT1* RNAi lines, in contrast, axillary buds did not show a clear photoperiodic response (Fig. 4C–F; Supplementary Fig. S5). In all RNAi lines, roughly two-thirds of axillary buds differentiated to runners and only very few buds produced branch crowns in both photoperiods. Moreover, in H4 and all transgenic lines, ~20–30% of axillary buds remained dormant (data not shown).

**Fig. 5.**
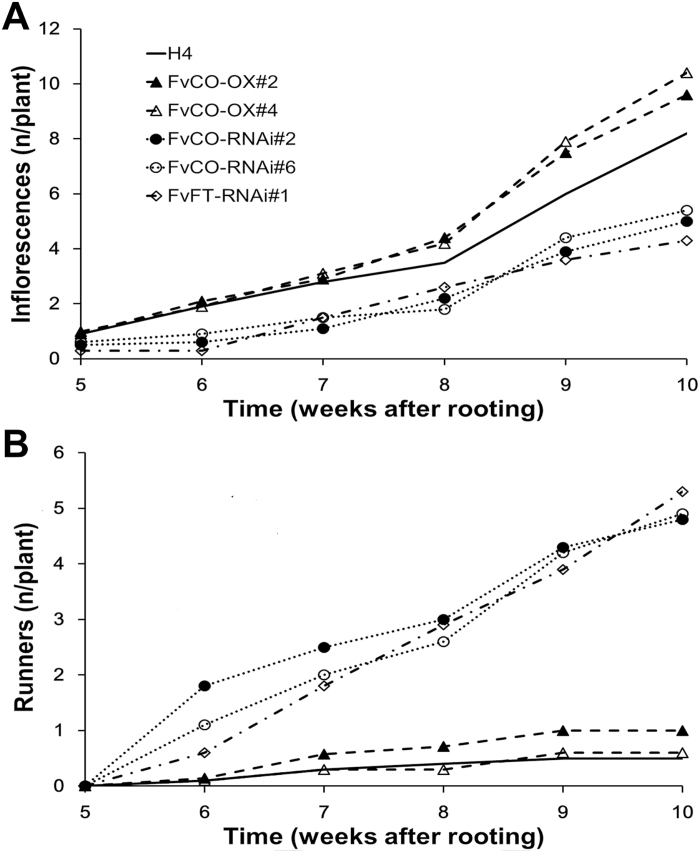
FvCO controls the balance between vegetative and generative development. Cumulative number of inflorescences (A) and runners (B) in clonally propagated plants of H4 and the indicated *FvCO* and *FvFT1* transgenic lines grown under LD conditions (*n*=10). To obtain generative plant materials in both wild-type H4 and transgenic lines, runner cuttings of flowering plants were rooted.

To explore further the effect of FvCO on the balance between generative and vegetative development, we observed the cumulative number of inflorescences and runners in generative plant materials. *FvCO* overexpression plants produced slightly more new inflorescences than the wild type (Fig. 5A). In *FvCO* and *FvFT1* RNAi plants, however, inflorescence production was reduced so that by the end of the experiment they had almost 50% fewer inflorescences than the H4 accession. In contrast to the intense flowering, runner production was strongly suppressed in overexpression and wild-type plants, whereas all RNAi lines continuously produced new runners at the rate of approximately one runner per week (Fig. 5B).

### *FvCO* up-regulates *FvFT1* in light

**Fig. 6.**
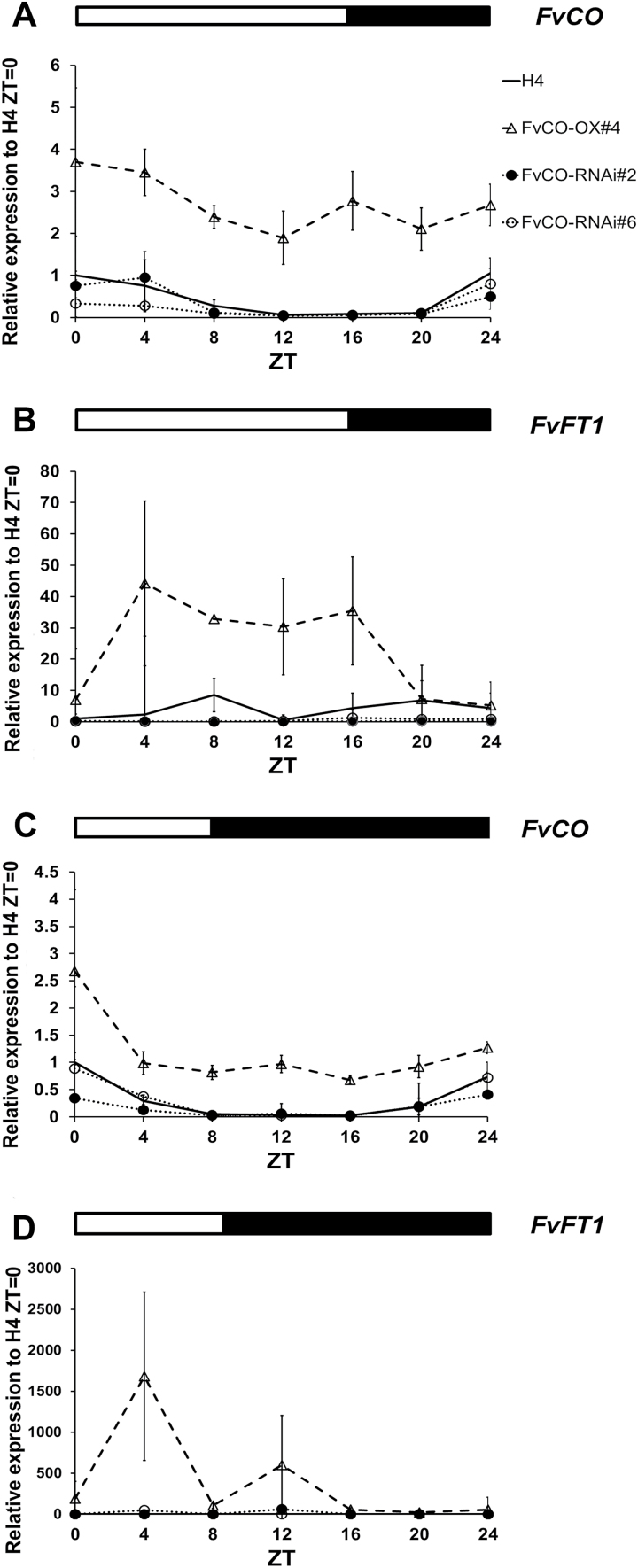
FvCO activates *FvFT1* in light. Diurnal expression of *FvCO* (A, C) and *FvFT1* (B, D) in the leaves of H4 and the indicated *FvCO* transgenic lines grown under LD (A, B) or SD (C, D) conditions. White and black bars above the panels indicate light and dark periods, respectively. The average expression level of three biological replicates is shown for each time point, all normalized to the expression level of *FvMSI1*, and the average of H4 ZT=0 is set as 1. Error bars indicate the SA. ZT, time (h) after dawn.

Next, we examined the expression of flowering time genes in *FvCO* transgenic lines. First, leaf samples were collected 4 h or 16 h after dawn (ZT=4 or 16) under LD conditions, as the *FvFT1* mRNA level peaks at these times in wild-type plants (Fig. 2B). The up-regulation of *FvFT1* was observed at both time points in *FvCO* overexpression lines (Supplementary Fig. S6). In RNAi lines, however, *FvFT1* mRNA expression was not detected. To understand the regulation of *FvFT1* by FvCO in more detail, we explored diurnal expression patterns in H4 and *FvCO* transgenic lines grown under LD and SD conditions. Overexpression of *FvCO* induced expression of *FvFT1* under both LD and SD conditions, but the normal diurnal expression cycle was lost (Fig. 6). Under LDs, up-regulation of *FvFT1* was observed during the light period from ZT0 to ZT16 in overexpression plants (Fig. 6A, B), while under SDs a strong up-regulation was observed only at ZT4 and another minor peak was present 4 h after dusk at ZT12 (Fig. 6C, D). In *FvCO* RNAi plants, in contrast, *FvFT1* mRNA levels remained extremely low or undetectable during the whole diurnal cycle under SD and LD conditions (Fig. 6B, D), even under continuous light which strongly increased *FvFT1* mRNA levels (Supplementary Fig. S7). These data indicated that FvCO affected both morning and evening peaks in *FvFT1* expression, even though *FvCO* expression was high only around dawn. Moreover, *FvFT1* expression is dependent on the light/dark cycle also in *FvCO* overexpression lines that highly express *FvCO* mRNA throughout the day.

**Fig. 7.**
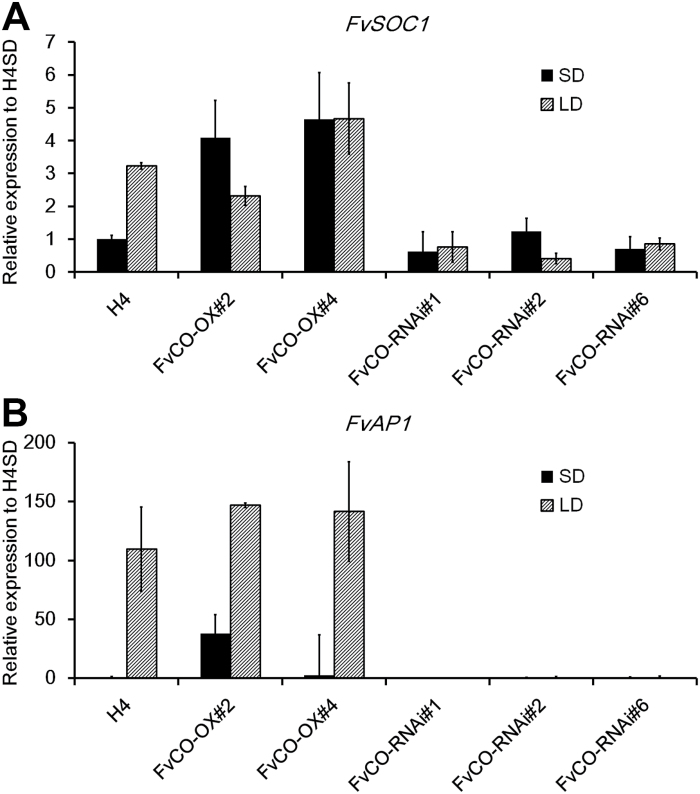
FvCO activates *FvSOC1* and *FvAP1* in the shoot apex. The expression of *FvSOC1* (A) and the floral meristem identity gene *FvAP1* (B) in shoot apices of *FvCO* transgenic lines and H4 control plants grown under SD or LD conditions for 3 weeks. The average expression level of three biological replicates is shown, all normalized to the expression level of *FvMSI1*, and H4 SD is set as 1. Error bars indicate the SD.

To explore further the downstream flowering gene pathway, we studied the expression of *FvSOC1*, that is activated by FvFT1 in shoot apices in LDs (Mouhu *et al.*, 2013), and the expression of the floral meristem identity gene *FvAP1*. *FvSOC1* was strongly activated in *FvCO* overexpression lines compared with H4 especially under SD conditions (Fig. 7A). In RNAi lines, however, the *FvSOC1* mRNA level was reduced in LD conditions and, in contrast to wild-type H4, no clear photoperiodic regulation of the gene was observed. Consistent with the observed differences in flowering time, *FvAP1* was down-regulated in RNAi lines and highly activated in the stronger SD-grown *FvCO* overexpression line compared with H4 at 3 weeks after the beginning of the treatment (Fig. 7B). However, an equally high *FvAP1* expression level was detected in wild-type and overexpression lines in LDs at this time point, but in another experiment, at a 1 week earlier time point, an elevated *FvAP1* expression level was detected in overexpression lines compared with H4 in LDs (Supplementary Fig. S8).

### *FvGI* and *FvFKF1* expression peaks precede the up-regulation of *FvFT1* towards evening in LDs

**Fig. 8.**
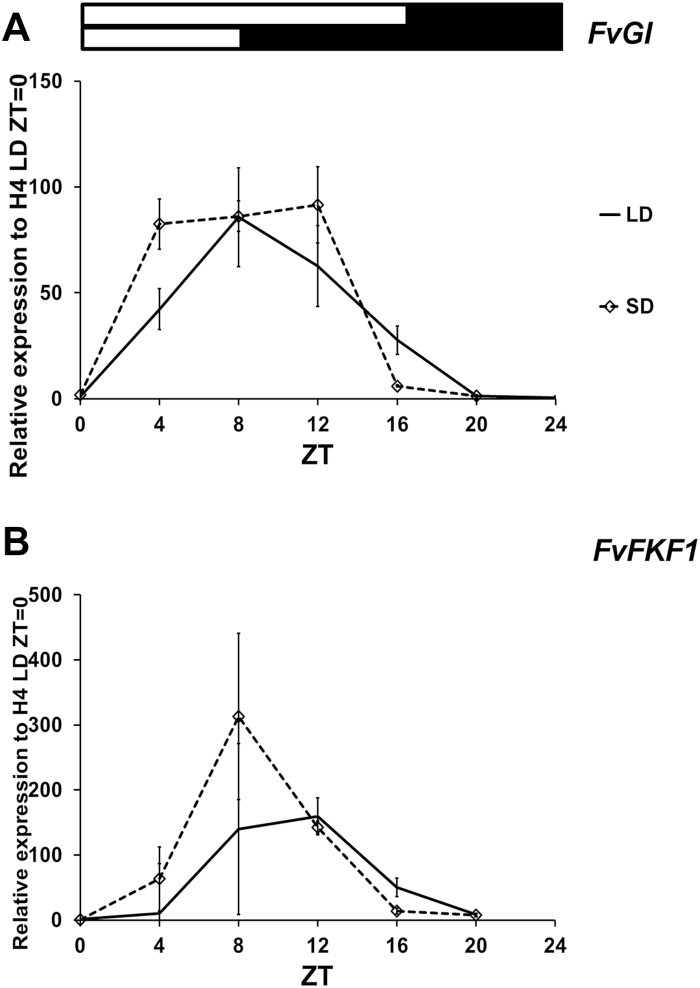
Expression patterns of *FvGI* and *FvFKF1*. Diurnal expression of *FvGI* (A) and *FvFKF1* (B) in the leaves of H4 under LD or SD conditions. White and black bars above the panels indicate light and dark periods, respectively. The average expression level of three biological replicates is shown for each time point, all normalized to the expression level of *FvMSI1*, and the average of H4 ZT=0 is set as 1. Error bars indicate the SD. ZT, time (h) after dawn.

Although *FvCO* is clearly required for the activation of *FvFT1* mRNA expression, additional factors are probably needed to schedule its diurnal cycle, especially towards evening (Fig. 2). Therefore, we studied the diurnal expression patterns of strawberry homologues of *GI* and *FKF1*, genes which encode regulators of *FT* expression in Arabidopsis (Sawa *et al.*, 2007; Sawa and Kay, 2011). In the H4 accession under 12 h SDs, the expression of *FvGI* increased rapidly in the morning and stayed high until ZT12, after which time there was a rapid drop in expression (Fig. 8A). Slightly slower up-regulation was observed under 16 h LD condidions, and *FvGI* expression remained high until dusk at ZT16; a similar expression pattern was also observed in FIN56 (Supplementary Fig. S9A). The expression of *FvFKF1* began to increase in the morning and peaked 8–12 after dawn (Fig. 8B; Supplementary Fig. S9B). The up-regulation was slower under LDs, where the strong activation took place between ZT4 and ZT8. The peak of expression of both *FvGI* and *FvFKF1* therefore preceded the up-regulation of *FvFT1* that takes place after ZT12, in the evening.

## Discussion

Plants typically contain a large *COL* gene family; for example Arabidopsis and rice have 17 and 16 genes, respectively, while 26 genes have been identified in soybean (Griffiths *et al.*, 2003; Wu *et al.*, 2014). A few of these genes encode floral activators, but also repressors as well as regulators, with no effect on flowering (Putterill *et al.*, 1995; Wong *et al.*, 2014; Cao *et al.*, 2015; Mulki and von Korff, 2016; Tan *et al.*, 2016). Here, we have identified 10 *COL* genes in woodland strawberry and shown that, based on phylogenetic analysis (Fig. 1; Supplementary Fig. S1), the previously identified *FvCO* is the only Group Ia *COL* gene in the *F. vesca* genome (Shulaev *et al.*, 2011). We have also shown that it plays a major role in the photoperiodic control of reproductive and vegetative development in this species. Although *FvCO* mRNA is expressed at different phases during the day compared with Arabidopsis *CO*, it is nevertheless required to generate the evening expression peak of *FvFT1* (a feature similar to the expression pattern of Arabidopsis *FT*; see Fig. 6; Suárez-López *et al.*, 2001), as well as an additional peak in the morning.

### FvCO controls photoperiodic flowering in strawberry

Previous studies suggested that the LD-activated *FvFT1*–*FvSOC1*–*FvTFL1* pathway represses flowering in woodland strawberry, and flower induction occurs after the silencing of this pathway by SDs and cool temperature in autumn. However, the characterization of the LD-flowering mutant H4, that is lacking the functional floral repressor FvTFL1, revealed a relic function of FvFT1 and FvSOC1 as floral activators in this accession (Koskela *et al.*, 2012; Mouhu *et al.*, 2013; Rantanen *et al.*, 2014, 2015). We show here that, similarly to the RNA silencing of *FvFT1*, the silencing of *FvCO* delays flowering in H4, while *FvCO* overexpression has an opposite effect (Figs 3, 4; Koskela *et al.*, 2012; Rantanen *et al.*, 2014). In agreement with these phenotypic observations, the silencing of *FvCO* strongly reduces *FvFT1* mRNA level in leaves, whereas *FvCO* overexpression leads to the activation of *FvFT1*. As previously observed in *FvFT1* RNAi lines (Mouhu *et al.*, 2013), in our *FvCO* transgenic lines, *FvSOC1* and *FvAP1* mRNA levels in shoot apices correlated positively with *FvFT1* expression in leaves. This indicates that in H4, *FvCO* has a major role in regulating *FvFT1* and *FvSOC1* expression to advance flowering under LD conditions. Also SD genotypes of woodland strawberry and cultivated strawberry may contain the relic flowering-promoting *FvCO*–*FvFT1*–*FvSOC1* pathway, but the activation of *FvTFL1* by FvSOC1 probably reverses the developmental outcome, namely the photoperiodic flowering response (Mouhu *et al.*, 2013; Koskela *et al.*, 2016). Direct functional analyses of *FvCO* and *FvFT1* in an SD genotype, however, are needed to confirm this model.

A recent study has suggested that another *FT*, *FaFT3*, is activated before *FaAP1* and may induce flowering in cultivated strawberry under SDs (Nakano *et al.*, 2015). A similar SD-specific activator may also function in the LD accession H4 which will eventually flower under SD conditions, when *FvFT1* expression is undetectable (Fig. 6D; Rantanen *et al.*, 2014). However, we found very low *FvFT3* expression in both H4 and *FvCO* transgenic lines in both SDs and LDs (data not shown). Thus, our results do not support the role of *FvFT3* in flower induction in H4.

Phylogenetic analysis grouped FvCO with other Group Ia COL proteins including Arabidopsis COL1 and COL2 that have no effect on flowering time (Ledger *et al.*, 2001; Kim *et al.*, 2013) and the major floral activator CO that has evolved from *COL1* or *COL2* by gene duplication in the Brassicaceae (Simon *et al.*, 2015). Unlike FvCO, studies on Group Ia COLs of the SD plant *P. nil* and the LD plant *M. truncatula* suggested that they do not promote flowering (Hayama *et al.*, 2007; Wong *et al.*, 2014); in *Glycine max*, COL1 functions as a floral repressor under LDs (Cao *et al.*, 2015). In the monocots rice and spring barley, however, the closest CO homologues Hd1 and HvCO2, respectively, activate flowering (Izawa *et al.*, 2002; Mulki and von Korff, 2016). This indicates that the functions of Group Ia COLs are species specific. What causes these diverse functions of CO homologues in flowering time regulation is an interesting open question.

### FvCO controls vegetative development in strawberry

Differentiation of strawberry axillary buds to runners and branch crowns is also regulated by photoperiod (Hytönen *et al.*, 2004). Our data demonstrate the major role of the FvCO/FvFT1-mediated photoperiodic pathway in this response as well as in controlling the balance between vegetative and floral development. H4 produced far more runners under SDs than under flower-inducing LDs, while the silencing of either *FvCO* or *FvFT1* caused continuous photoperiod-independent production of runners. *FvCO* overexpression plants, however, produced slightly fewer runners than H4 and, when these plants were moved from SDs to flower-inductive LD conditions, their runner production slowed down earlier than in H4. LD, in contrast, promoted the differentiation of axillary buds to branch crowns in H4 and *FvCO* overexpression lines, whereas RNAi lines did not show this response.

In contrast to runner production, generative *FvCO* RNAi plants produced fewer and overexpression lines slightly more inflorescences than H4 (Fig. 5). Such a balance between vegetative and generative growth is well documented in cultivated strawberries (e.g. Sønsteby and Heide, 2007), and it may be caused by competition for resources in clonal plants (Loehle, 1987). Furthermore, *FvCO* and its counterpart in cultivated strawberry can affect the expression/function of the gene at the *PFRU* locus that has been reported to control the balance between vegetative and generative growth (Gaston *et al.*, 2013; Samad *et al.*, 2017).

In the SD accession of woodland strawberry, FvSOC1 promotes runner formation in LDs (Mouhu *et al.*, 2013), and studies in non-flowering *FvTFL1* overexpression plants and in a non-transgenic SD cultivar of cultivated strawberry confirmed that direct photoperiodic regulation of axillary bud differentiation can occur (Hytönen *et al.*, 2009; Koskela *et al.*, 2012). In H4 and *FvCO* transgenic lines, however, we found a negative correlation between the *FvSOC1* expression level and the number of runners. Therefore, our data suggest that in this accession, which flowers perpetually after flower induction, axillary bud differentiation is primarily controlled by flowering, and FvSOC1 may have a minor role. Runners are formed from axillary buds at the vegetative stage and, upon flower induction, the uppermost axillary buds differentiate into new branch crowns instead of runners, which leads to a reduction in runner formation and increases the number of meristems that can produce inflorescences (Hytönen *et al.*, 2004). Taken together with this information, our study indicates that the photoperiodic pathway affects the balance between vegetative and generative development in strawberries; further studies are needed to uncover how this balance is regulated in LD and SD genotypes.

### The diurnal *FvFT1* expression is under control of FvCO

In Arabidopsis, the *CO* mRNA level increases towards evening and, according to the external coincidence model, *FT* is activated under LDs when *CO* expression coincides with light (Suárez-López *et al.*, 2001). Similarly to Arabidopsis *FT*, *FT* homologues in woodland strawberry and cultivated strawberry (*FvFT1* and *FaFT1*, respectively), exhibited a major mRNA expression peak in the evening at ZT16–ZT20 (Fig. 6; Koskela *et al.*, 2012, 2016). However, an additional peak was observed between 4 h and 8 h after dawn; other work shows that the height of this peak depends on the light conditions (Rantanen *et al.*, 2014). *FvCO* is expressed in a different phase from Arabidopsis *CO* in both LD and SD accessions (Supplementary Fig. S2; Kurokura, 2009). It exhibits a sharp expression peak towards dawn, similar to *COL1*, *COL2*, and *COL5*, *BvCOL1* of *Beta vulgaris*, and *PnCO* of *P. nil* (Ledger *et al.*, 2001; Liu *et al.*, 2001; Chia *et al.*, 2008; Hassidim *et al.*, 2009). The dawn signal (dark to light) causes the down-regulation of the *COL* gene in the SD plant *Chenopodium rubrum* (Drabešová *et al.*, 2014), and this is also likely to be the case in woodland strawberry, because the transfer of plants to darkness caused accumulation of *FvCO* mRNA after subjective dawn (Fig. 2c).

Our studies on transgenic lines indicate that, although diurnal expression rhythms of *FvCO* and *FvFT1* do not match in woodland strawberry, functional FvCO is needed to activate *FvFT1* mRNA expression in both the morning and evening in LDs. *FvCO* RNAi lines exhibit very low *FvFT1* mRNA levels during the whole diurnal cycle compared with the wild type, whereas overexpression of *FvCO* results in the induction of *FvFT1* in a light-dependent manner with a broad peak during the light period under LDs. Under SDs, however, *FvFT1* is highly activated only in the morning in overexpression plants, with an additional minor peak after dusk (Fig. 6). Our results in *FvCO* overexpression plants suggest that the FvCO protein is regulated by light, as has been observed in Arabidopsis (Valverde *et al.*, 2004; Song *et al.*, 2012). Although the *FvCO* expression pattern is different from that of *CO* (Suárez-López *et al.*, 2001), light-regulated FvCO protein could form a part of the photoperiod measurement system that controls the gradual up-regulation of *FvFT1* under increasing photoperiods (Rantanen *et al.*, 2015). However, additional unknown factors are probably needed to schedule the evening peak of *FvFT1*. These factors may include CRYPTOCHROME-INTERACTING BASIC-HELIX-LOOP-HELIX and/or PHYTOCHROME-DEPENDENT LATE-FLOWERING proteins that activate *FT* specifically in the evening (Endo *et al.*, 2013; Liu *et al.*, 2013). Further studies on these regulators as well as on FvCO protein stability and activity are needed to understand the photoperiodic control of *FvFT1* mRNA expression.

In Arabidopsis, GI and FKF1 interact in a blue light-dependent manner to activate *CO* and *FT* mRNA expression by removing the repressor protein CDF1 in the afternoon (Imaizumi *et al.*, 2005; Sawa *et al.*, 2007; Fornara *et al.*, 2009; Song *et al.*, 2012). In addition, FKF1 and GI can directly activate the expression of *FT* (Sawa and Kay, 2011). Since *CO*-independent *FT* regulation has also been suggested in other species (Doi *et al.*, 2004; Hayama *et al.*, 2007; Ridge *et al.*, 2016), it is unlikely that *FvFT1* expression is regulated only by *FvCO* in woodland strawberry, even though *FvCO* seems to play a major role.

To gain insight into the function of these genes in woodland strawberry, we investigated their diurnal expression rhythms and observed that *FvGI* was highly expressed during the day in both SDs and LDs (Fig. 8A). *FvFKF1* exhibited a sharper expression peak in the afternoon, a few hours before the *FvFT1* evening peak (Fig. 8B). Therefore, FvFKF1 and FvGI may control the expression of *FvFT1* in the evening in LDs, but detailed gene functional studies are needed to confirm their roles in the photoperiodic flower induction of the woodland strawberry.

### Conclusions

The *CO* homologue of the woodland strawberry, *FvCO*, has a diurnal expression rhythm with a sharp peak around dawn, regardless of photoperiodic conditions. FvCO plays a major role in the photoperiodic regulation of *FvFT1* and thus flowering time, as well as in vegetative reproduction (i.e. the production of runners). The expression of *FvCO* is promoted under darkness, and light is required to suppress its expression in the morning. Partial coincidence of the expression pattern of *FvCO* and *FvFT1* in the morning indicates that *FvCO1* regulates *FvFT1* expression in part, but other unknown factor(s) may be involved in the generation of the bimodal diurnal expression pattern of *FvFT1*. Woodland strawberry homologues of FvGI and FvFKF1 are good candidates for the factors that schedule *FvFT1* expression in the evening because, as in Arabidopsis, corresponding genes are expressed during the day before the *FvFT1* evening peak.

## Supplementary data

Supplementary data are available at *JXB* online.

Fig. S1. Full structure of the phylogenetic tree of COL proteins.

Fig. S2. The analysis of conserved motifs of Group I COL proteins.

Fig. S3. Expression patterns of *FvCO* and *FvFT1* in FIN56.

Fig. S4. Expression patterns of *COL* genes 14981 and 27383 in H4 and *FvCO* RNAi lines.

Fig. S5. Vegetative and reproductive growth of *FvCO* transgenic lines under LD conditions.

Fig. S6. Expression of *FvFT1* in *FvCO* transgenic plants.

Fig. S7. *FvCO* and *FvFT1* expression under continuous light.

Fig. S8. *FvAP1* expression in *FvCO* transgenic plants.

Fig. S9. Expression patterns of *FvGI* and *FvFKF1* in SD accession FIN56.

Table S1. List of primers used in quantitative real-time PCR.

Table S2. List of protein accession numbers used in the phylogenetic tree.

Table S3. Flowering time of Hawaii-4 and *FvCO* transgenic lines.

## Supplementary Material

Supplementary_figures_S1_S9_and_tables_S1_S3
