# Supplementary material for: Fragaria vesca CONSTANS controls photoperiodic flowering and vegetative development
Source: J Exp Bot. 2017 Sep 25;68(17):4839–50. doi: 10.1093/jxb/erx301 (PMC5853477; doi:10.1093/jxb/erx301)
Supplement: Supplementary_figures_S1_S9_and_tables_S1_S3 [file erx301_suppl_supplementary_figures_s1_s9_and_tables_s1_s3.pdf]

Fragaria vesca **CONSTANS controls photoperiodic flowering and vegetative development**

Takeshi Kurokura, Samia Samad, Elli Koskela, Katriina Mouhu and Timo Hytönen

Supplementary data

Figure S1. Full structure of the phylogenetic tree of COL proteins.

Figure S2. The analysis of conserved motifs of group I COL proteins.

Figure S3. Expression patterns of *FvCO* and *FvFT1* in FIN56.

Figure S4. Expression patterns of *COL* genes 14981 and 27383 in H4 and *FvCO* RNAi lines.

Figure S5. Vegetative and reproductive growth of *FvCO* transgenic lines under LD conditions.

Figure S6. Expression of *FvFT1* in *FvCO* transgenic plants.

Figure S7. *FvCO* and *FvFT1* expression under continuous light.

Figure S8. *FvAP1* expression in *FvCO* transgenic plants.

Figure S9. Expression patterns of *FvGI* and *FvFKF1* in SD accession FIN56.

Table S1. List of primers used in quantitative real-time PCR.

Table S2. List of protein accession numbers used in the phylogenetic tree.

Table S3. Flowering time of Hawaii-4 and *FvCO* transgenic lines.

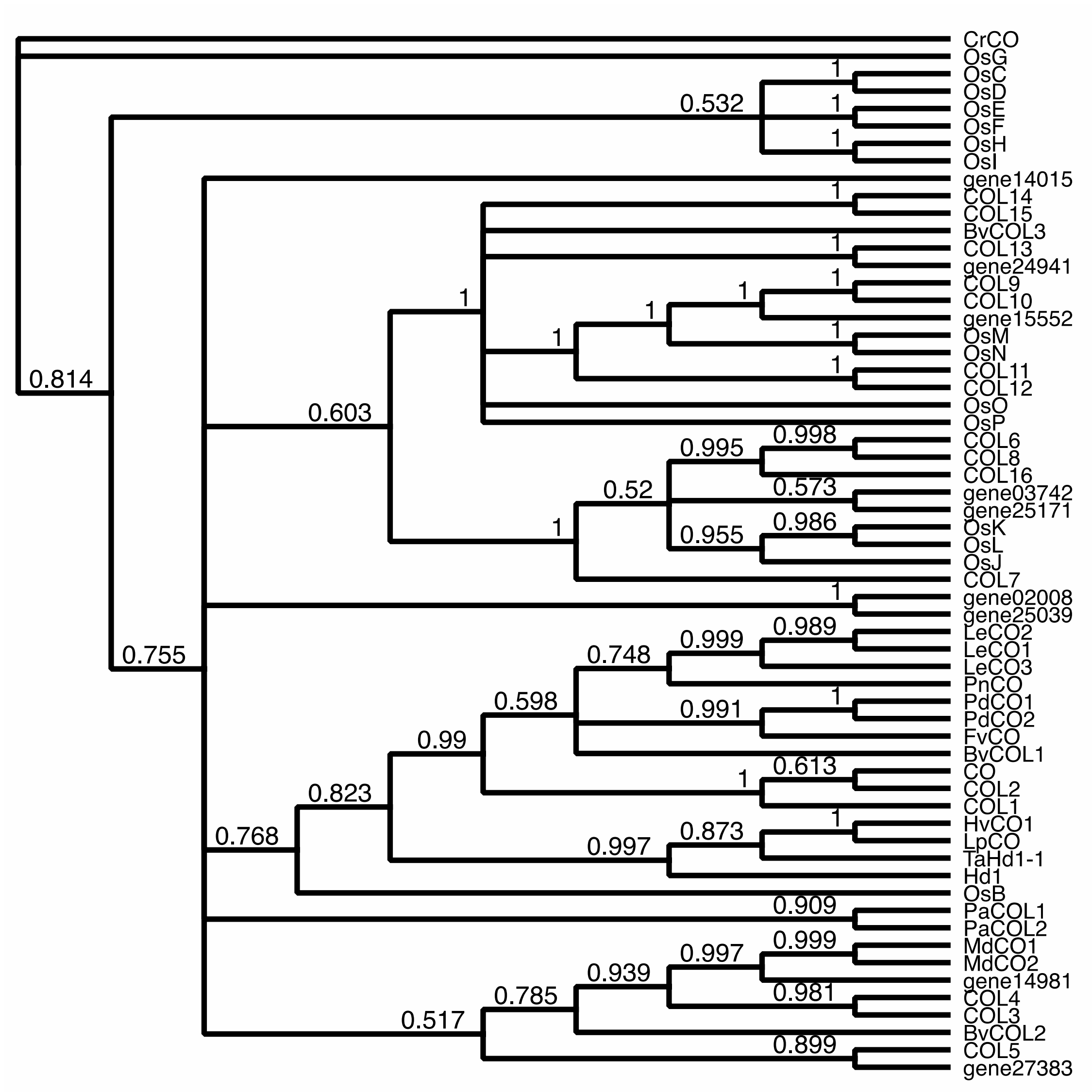

Supplementary Figure S1. Full structure of the phylogenetic tree of COL proteins. Sequence accession numbers are listed in Supplementary Table S2.

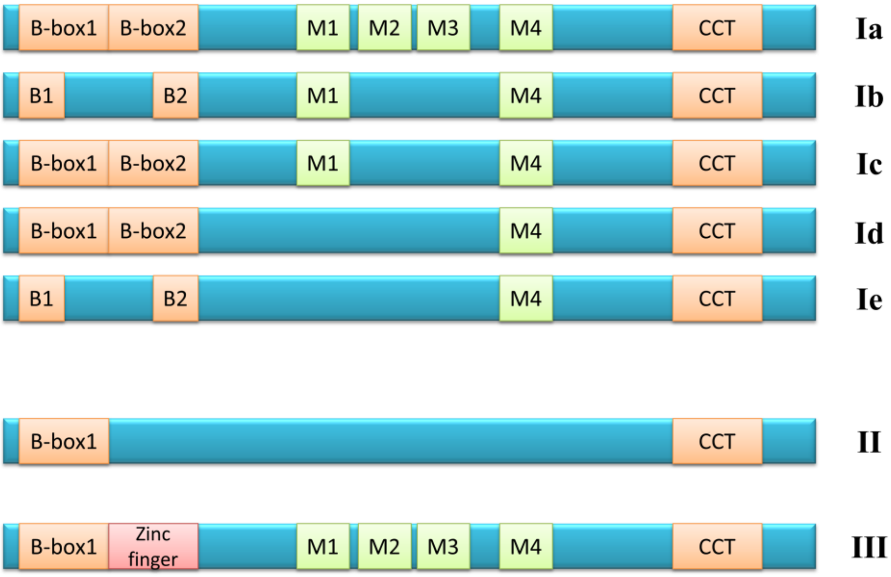

Supplementary Figure S2a Comparison of structures of COL proteins.

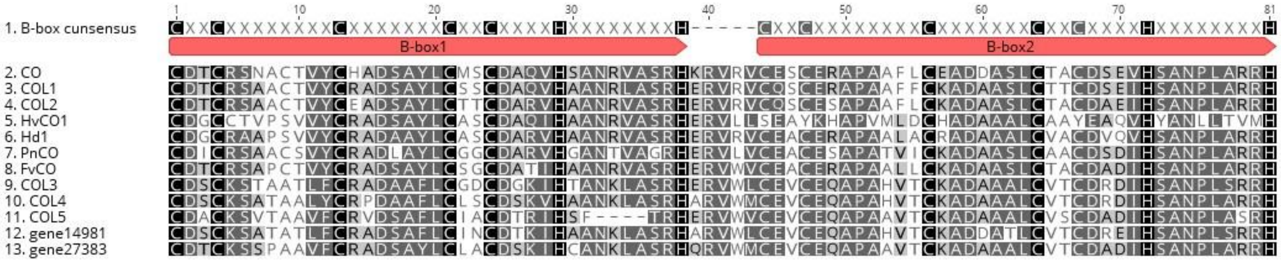

Supplementary Figure S2b. Alignment of B-boxes of COL Group I proteins.

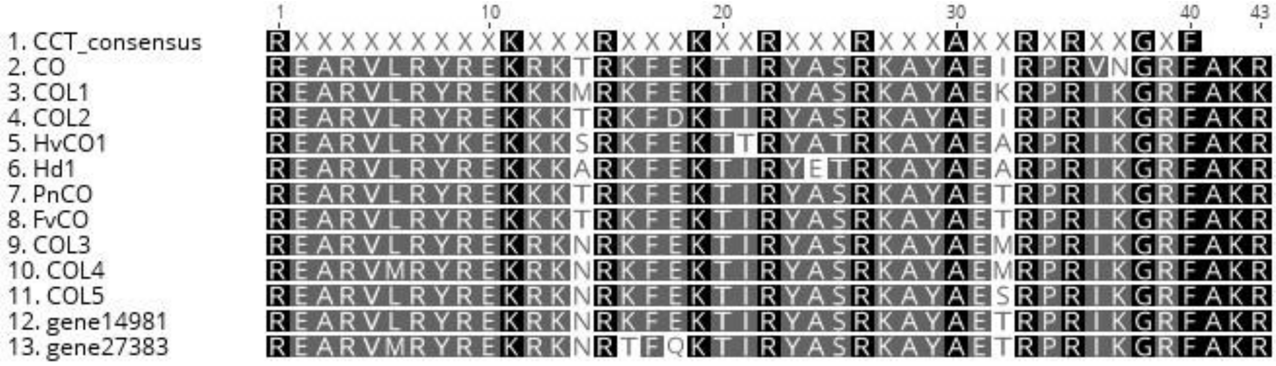

Supplementary Figure S2c. Alignment of CCT domain of COL Group I proteins.

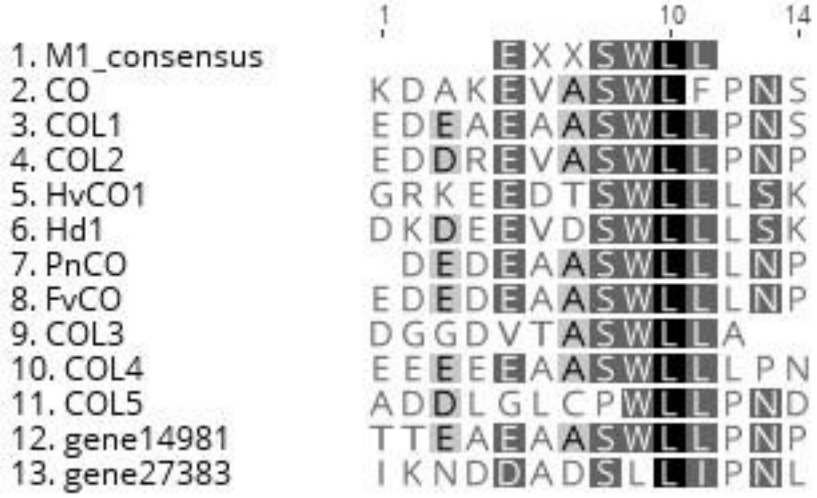

Supplementary Figure S2d. Alignment of M1 region of COL Group I proteins.

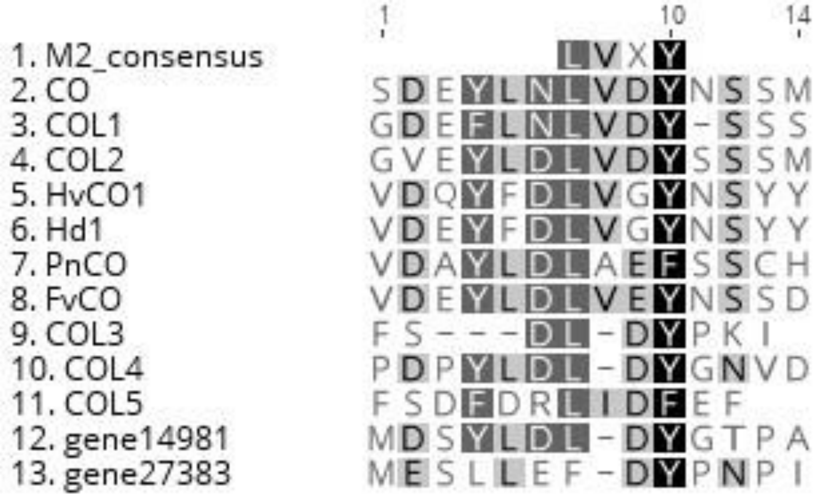

Supplementary Figure S2e. Alignment of M2 region of COL Group I proteins.

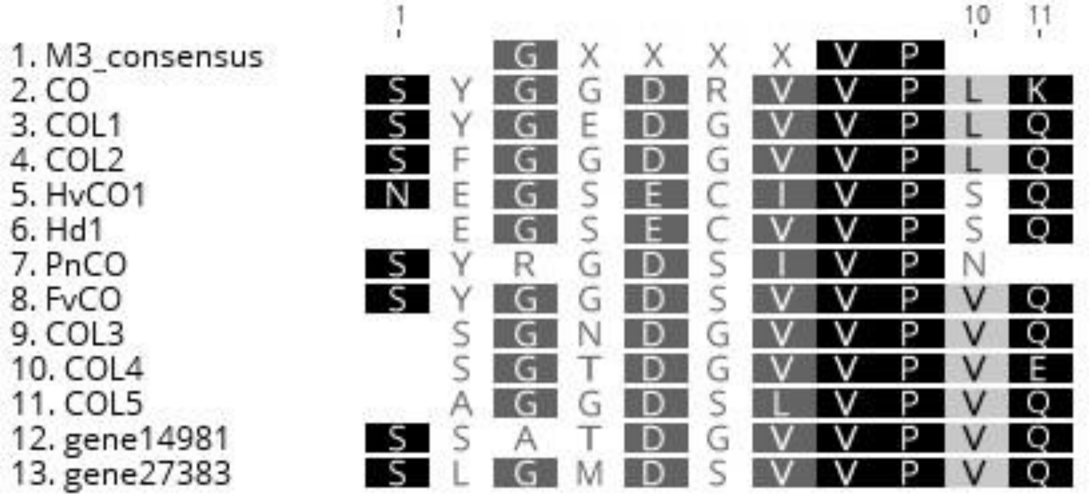

Supplementary Figure S2f. Alignment of M3 region of COL Group I proteins.

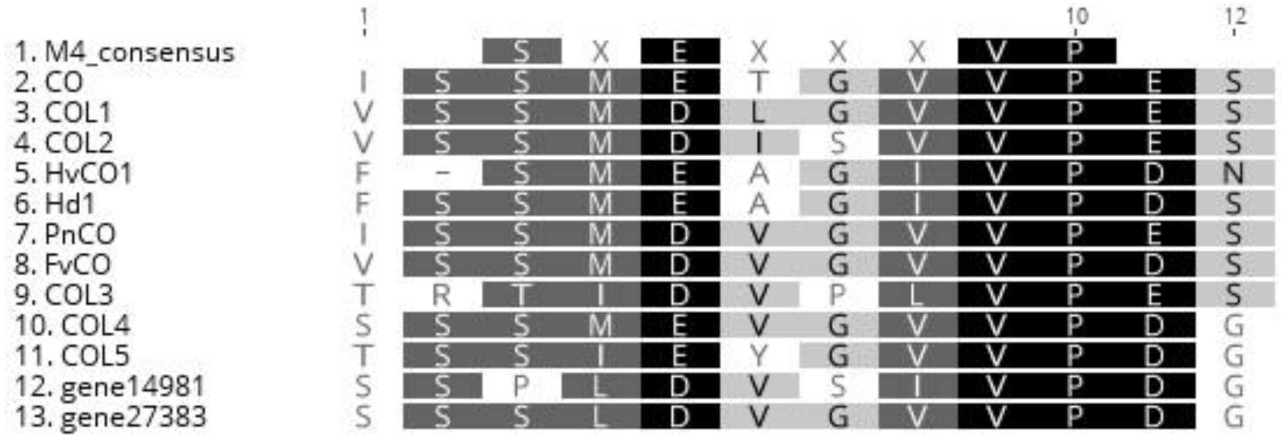

Supplementary Figure S2g. Alignment of M4 region of COL Group I proteins.

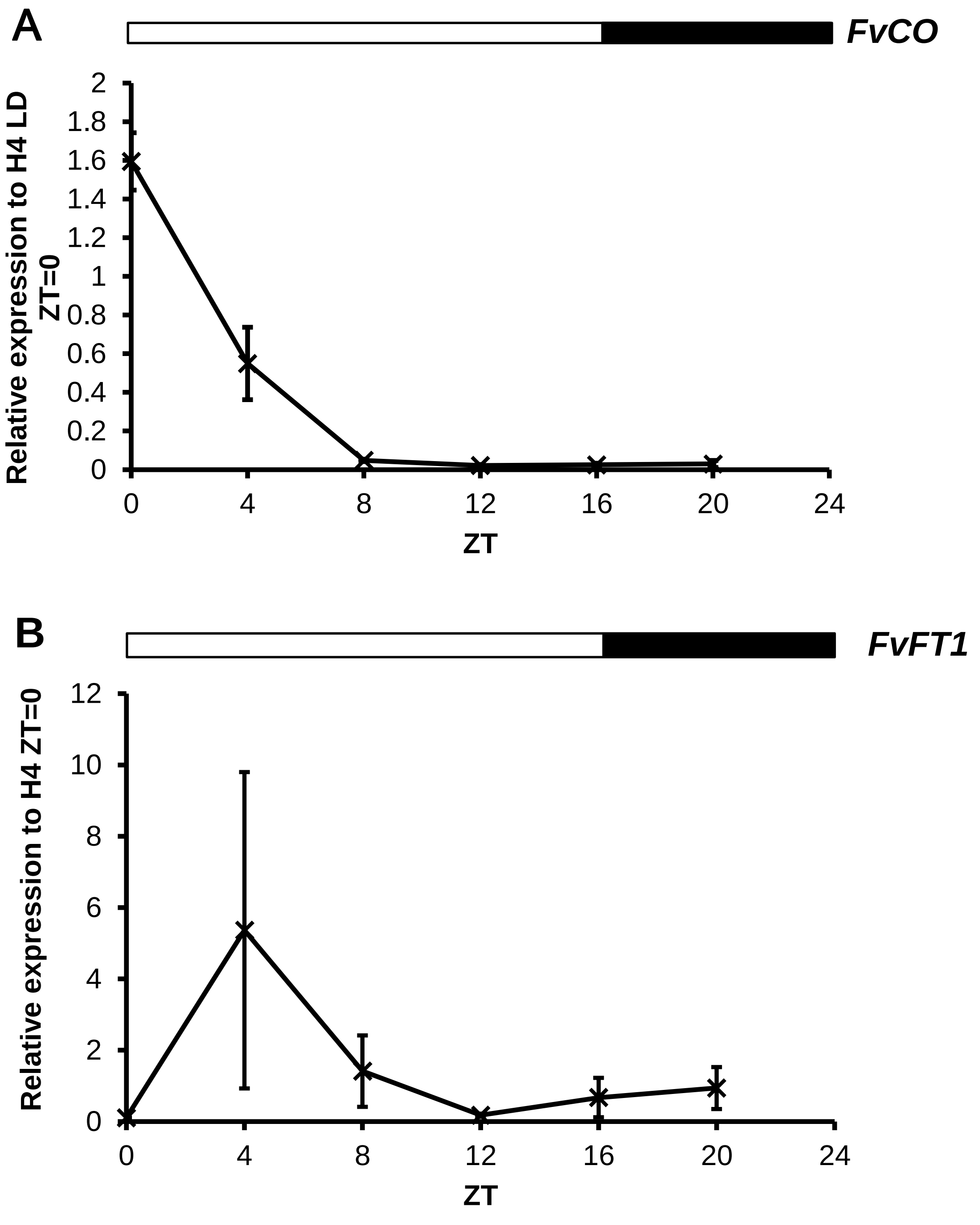

Supplementary Figure S3. Expression patterns of *FvCO* and *FvFT1* in FIN56.

mRNA expression patterns of *FvCO* (A) and *FvFT1* (B) were analyzed in the leaf samples of long-day (LD) grown FIN56 plants. White and black bars above the panels indicate light and dark periods, respectively. Average expression level of three biological replicates is shown for each time point, all normalized to the expression level of *FvMSI1*, and the average of H4 at ZT=0 under LD (Fig. 2A and B) is set as 1. Error bars indicate standard deviation.

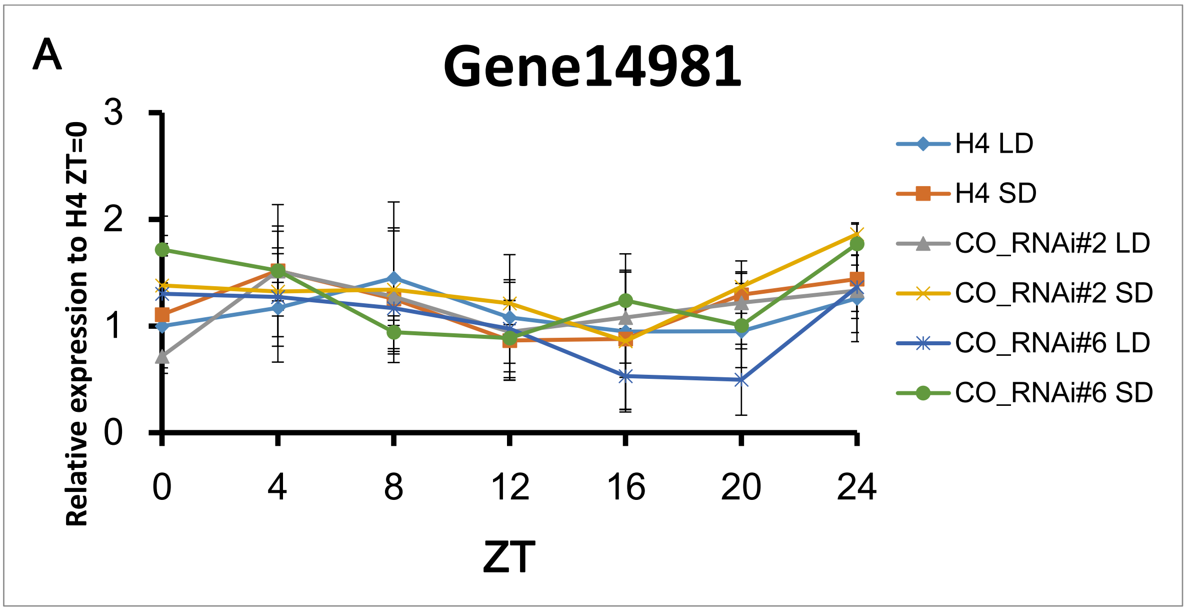

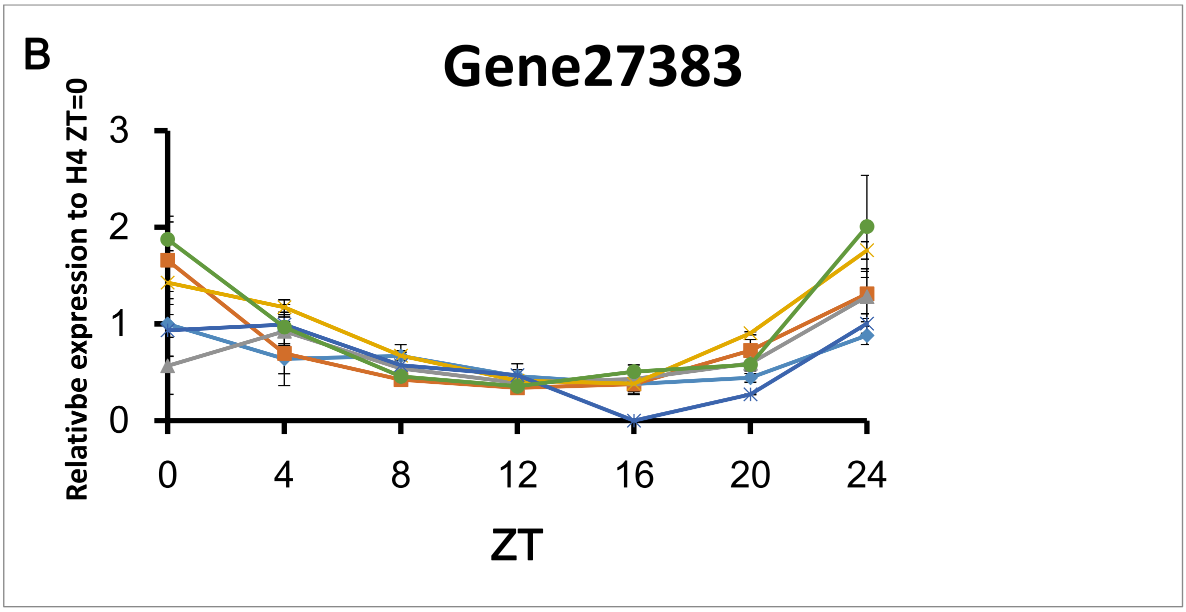

Supplementary Figure S4. Expression patterns of *COL* genes 14981 and 27383 in H4 and *FvCO* RNAi lines.

mRNA expression patterns of gene14981 (A) and gene27383 (B) were analyzed in the leaf samples of Hawaii-4 (H4) and *FvCO* RNAi plants grown under LD or SD conditions. Average expression level of three biological replicates is shown for each time point, all normalized to the expression level of *FvMSI1*, and the average of H4 ZT=0 under LD is set as 1. Error bars indicate standard deviation.

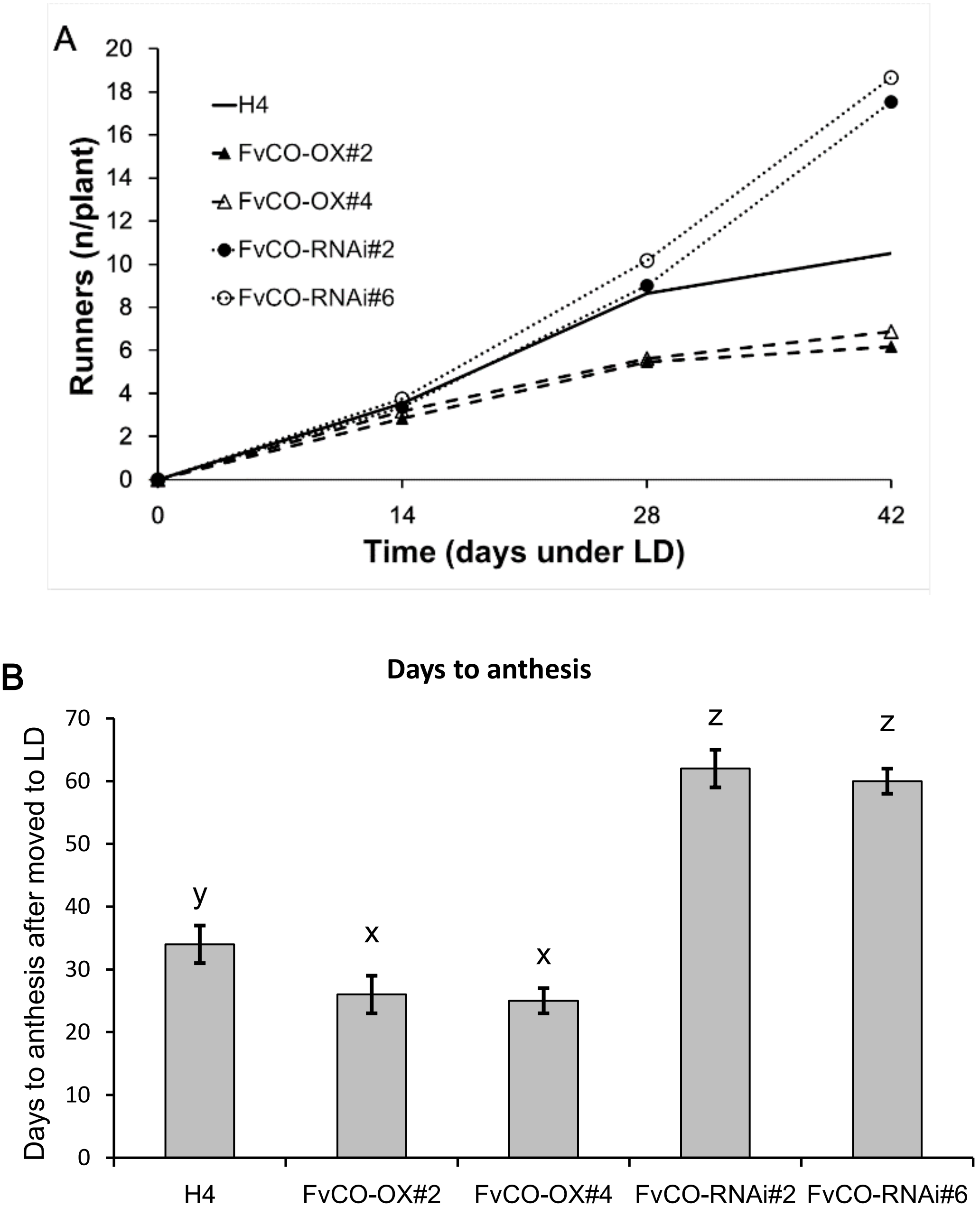

Supplementary Figure S5. Vegetative and reproductive growth of H4 and *FvCO* transgenic lines under LD conditions.

Cumulative number of runners in young seedlings of H4 and the indicated *FvCO* transgenic lines (A) and days to anthesis (B) are shown (*n*=13-17). Seedlings were raised under SD conditions, moved to LD at approximately 2-leaf stage, and the growth observations were carried out from the beginning of the LD treatment. Different letters indicate significant difference between the genotypes based on Tukey-Kramer test at $P<0.05$. Error bars indicate standard deviation.

A

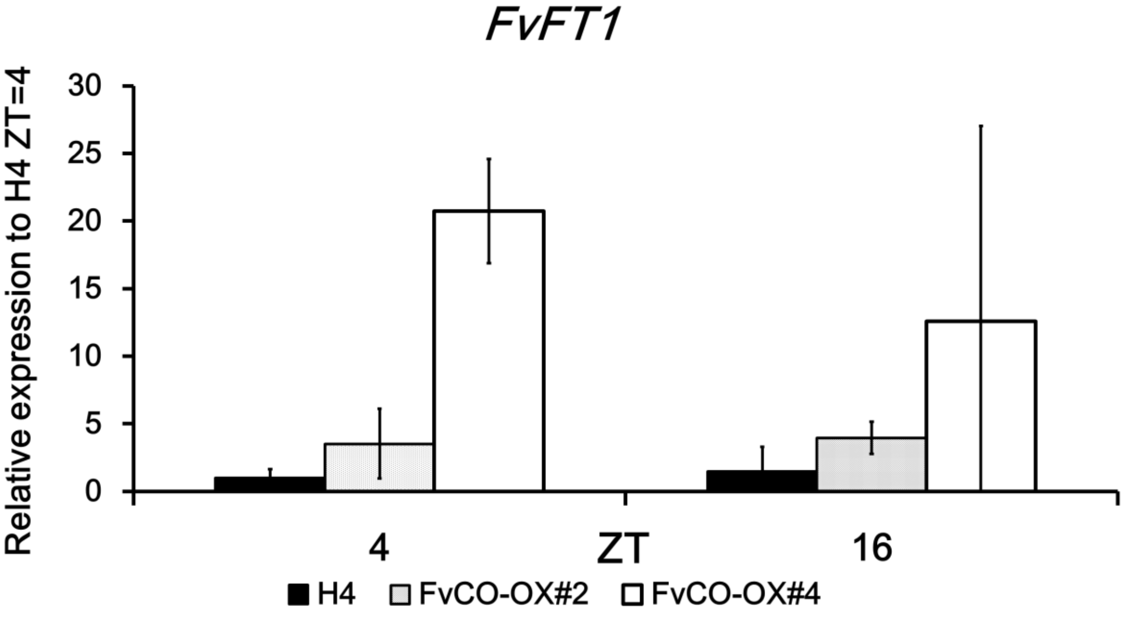

B

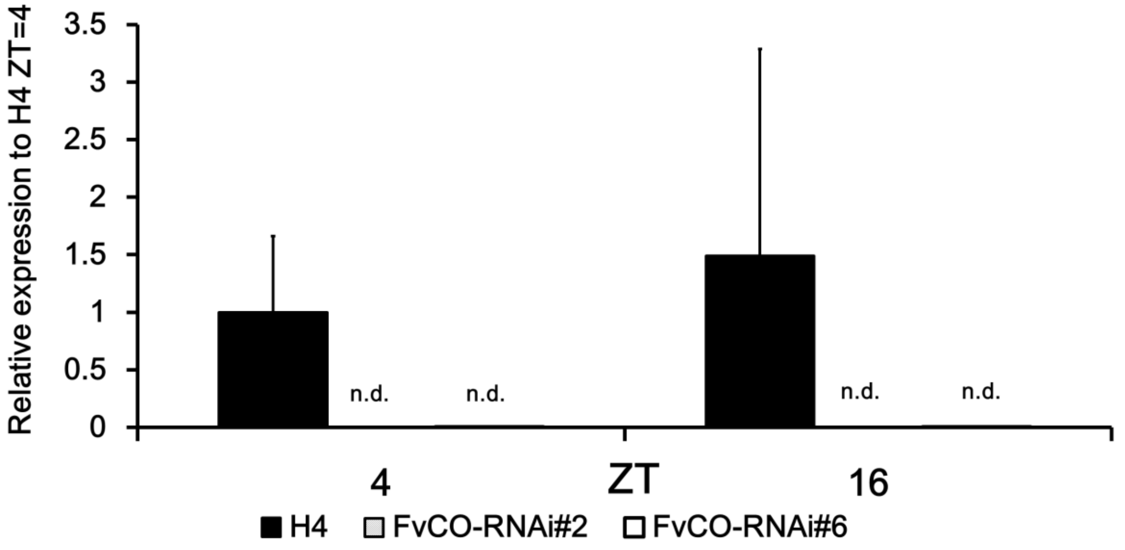

Supplementary Figure S6. Expression of *FvFT1* in *FvCO* transgenic plants.

The expression of *FvFT1* was analyzed in the indicated transgenic lines in LD using same samples as in Fig. 3a, b. Leaf samples were collected either four or 16 h after dawn (ZT4 and ZT16, respectively). Average expression level of three biological replicates is shown for each sampling point, all normalized to the expression level of *FvFMSI1*. Error bars indicate standard deviation. n.d. = not detected.

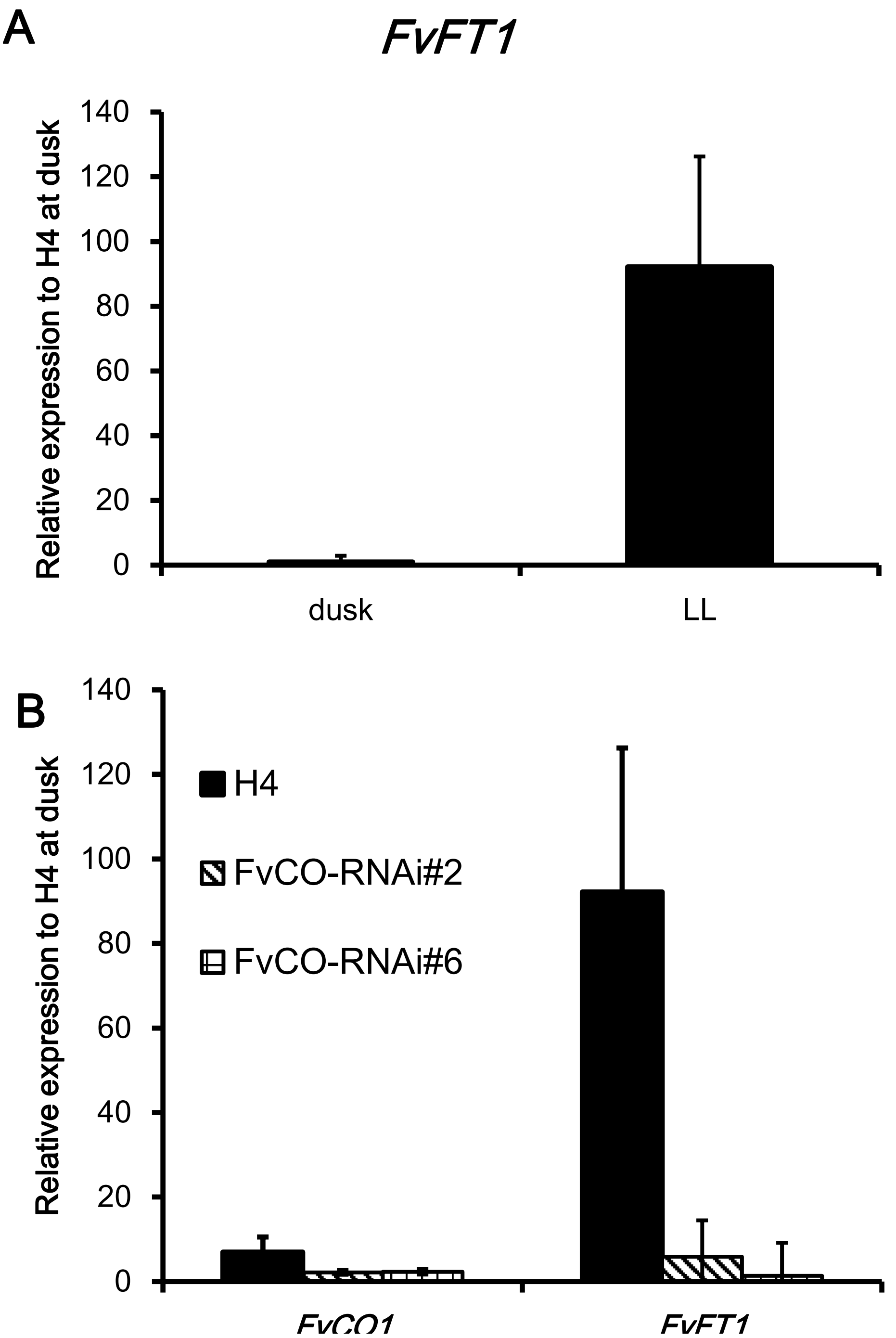

Supplementary Figure S7. *FvCO* and *FvFT1* expression under continuous light.

(A) Expression of *FvFT1* in the leaves of H4 at dusk in LD or at subjective dusk after one week of continuous light (LL). (B) Expression of *FvCO* and *FvFT1* in the leaves of H4 and *FvCO* RNAi lines grown under continuous light. Average expression level of three biological replicates is shown for each time point, all normalized to the expression level of *FvMSI1*, and the average of H4 at dusk is set as 1. Error bars indicate standard deviation.

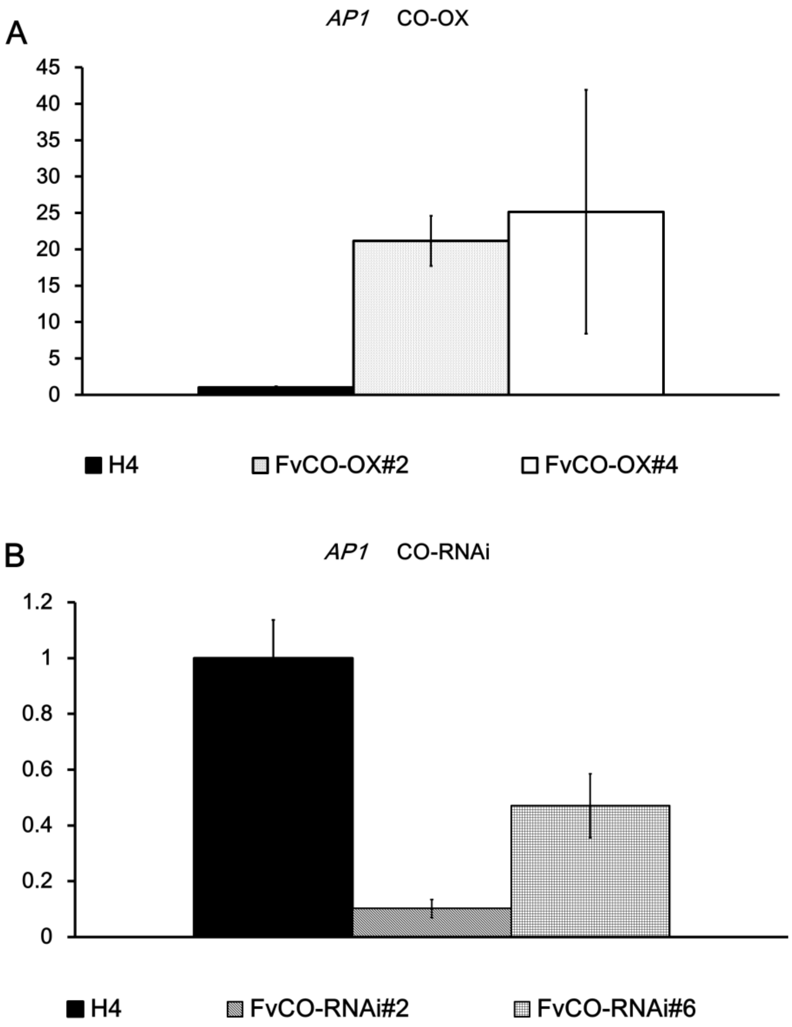

Supplementary Figure S8. *FvAP1* expression in *FvCO* transgenic plants.

The expression of *FvAP1* was analyzed in the shoot apices of H4 and indicated transgenic lines. Shoot apex samples were collected after plants were grown under LD conditions for two weeks. Average expression level of three biological replicates is shown for each sampling point, all normalized to the expression level of *FvFMSI1*. Error bars indicate standard deviation.

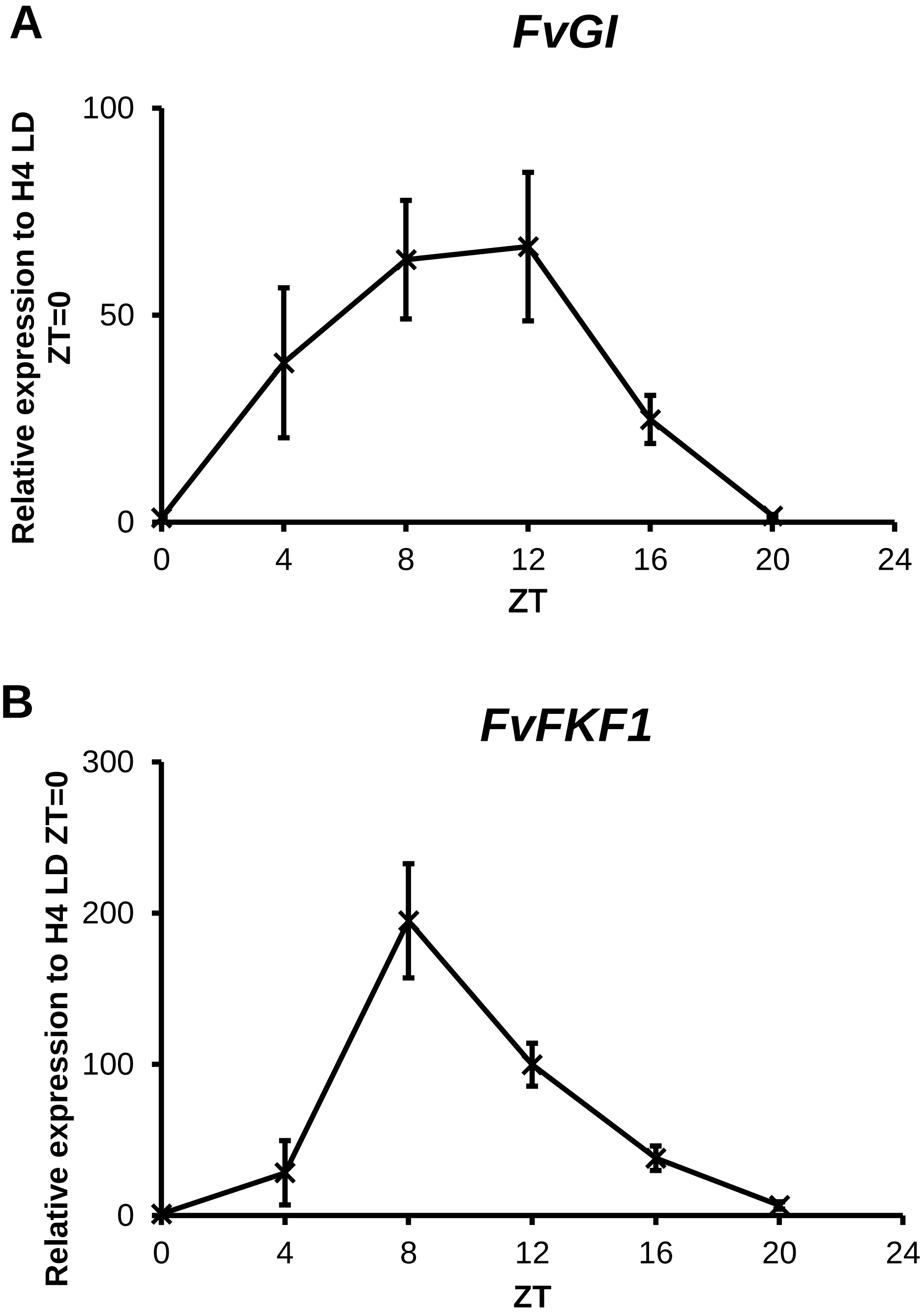

Supplemental Figure S9. Expression patterns of *FvGI* and *FvFKF1* in SD accession FIN56.

Diurnal expression of *FvGI* (A) and *FvFKF1* (B) in the leaves of FIN56 under LD conditions. White and black bars above the panels indicate light and dark periods, respectively. Average expression level of three biological replicates is shown for each time point, all normalized to the expression level of *FvMSI1*, and the average of H4 ZT=0 under LD (Fig. 6) is set as 1. Error bars indicate standard deviation. ZT = time (h) after dawn.

Supplementary Table S1. List of primers used in quantitative real-time PCR

Gene	Foward	Reverse
FvCO	5’-GACATCCACTCCGCCAAC-3’	5’-GTGGACCCCACCACTATCTG-3’
FvFT1	5’-CAATCTCTTGGCCGAAAACT-3’	5’-TGAGCTCAAACCTTCCCAAG-3’
FvAP1	5’-AGCTCAGGAGGTTCATGACTG-3’	5’-TAAGGTCGAGCTGGTTCCTC-3’
FvFUL1	5’-GCAGTGCATGAATCCCTTTC-3’	5’-GCTGGTGATTTTGGAGCTTG-3’
FvMSI1	5’-TCCCCACACCTTTGATTGCCA-3’	5’-ACACCATCAGTCTCCTGCCAAG-3’
FvUBQ	5’-CGCACCCTAGCAGACTACAA-3’	5’-GCAGAGTGCTCTCCTTCTGG-3’

Supplementary Table S2. List of protein accession numbers used in the phylogenetic tree.

Species	Gene name	Accession Number	Species	Gene name	Accession Number
A. thaliana	CO	NP_197088	*M. domestica*	MdCO1	AAC99309
	COL1	NP_197089		MdCO2	AAC99310
	COL2	NP_186887	*O. sativa*	Hd1a	ABB17665
	COL3	NP_180052		OsB	BAA33205
	COL4	NP_197875		OsC	CAE03116
	COL5	NP_568863		OsD	AAAA01000818
	COL6	NP_564932		OsE	BAD37550
	COL7	NP_177528		OsF	AAAA01022688
	COL8	NP_175339		OsG	AAAA01008321
	COL9	NP_187422		OsH	AAL79780
	COL10	NP_199636		OsI	BAC07164
	COL11	NP_193260		OsJ	AAX95654
	COL12	NP_188826		OsK	AAAA01010759
	COL13	NP_182310		OsL	BAD54363
	COL14	NP_973589		OsM	AAAA01003850
	COL15	NP_174126		OsN	BAA33206
	COL16	NP_173915		OsO	BAD01231
B. vulgaris	BvCOL1	ACC95129		OsP	BAA33200
	BvCOL2	ACC95129	*P. abies*	PaCOL1	CAK26139
	BvCOL3	ACC95131		PaCOL2	ABR57243
C. reinhardtii	CrCO	CAP74566	*P. deltoids*	PdCO	AAS00054
H. vulgare	HvCO1	AAM74062		PdCO2	AAS00055
L. esculentum	LeCOL1	AAS67376	*P. nil*	PnCO	AAG24863
	LeCOL2	AAS67378	*T. aestivum*	TaHd1-1	BAC92735
	LeCOL3	AAS67379			
L. perenne		CAH55695			

Supplementary Table S3. Flowering time of Hawaii-4 and *FvCO* transgenic lines.

The number of leaves before anthesis were counted under long and short days (LD and SD, respectively) at 22°C. Values are mean ± standard deviation (n=10). Values followed by different letters are significantly different from each other based on Tukey-Kramer test at P<0.05.

Genotype	Number of leaves	
	LD (16/8h)	SD (8/16h)
Hawaii-4	6.6±0.5 b	12.8±1.1 f
FvCO-OX#2	4.8±0.5 a	7.8±0.7 bc
FvCO-OX#4	5.1±0.6 a	8.6±0.9 c
FvCO-RNAi#2	10.7±0.9 d	13.3±0.8 f
FvCO-RNAi#6	11.4±1.0 de	13.1±0.6 f
FvFT1-RNAi#1	12.3±1.6 ef	12.8±0.8 f
